# The impact of reducing fatty acid desaturation on the composition and thermal stability of rapeseed oil

**DOI:** 10.1111/pbi.13263

**Published:** 2019-10-14

**Authors:** Harjeevan Kaur, Lihong Wang, Natalia Stawniak, Raymond Sloan, Harrie van Erp, Peter Eastmond, Ian Bancroft

**Affiliations:** ^1^ University of York Heslington York UK; ^2^ Biorenewables Development Centre Dunnington York UK; ^3^ Rothamsted Research Harpenden UK; ^4^ Present address: Punjab Agricultural University Ludhiana India

**Keywords:** *Brassica napus*, rapeseed, oilseed rape, polyunsaturated fatty acids, thermal stability, erucic acid

## Abstract

Oilseed rape (*Brassica napus*) is the third largest source of vegetable oil globally. In addition to food uses, there are industrial applications that exploit the ability of the species to accumulate the very‐long‐chain fatty acid (VLCFA) erucic acid in its seed oil, controlled by orthologues of *FATTY ACID ELONGASE 1* (*Bna.FAE1.A8* and *Bna.FAE1.C3*). The proportion of polyunsaturated fatty acids (PUFAs) in rapeseed oil is predicted to affect its thermal stability and is controlled by orthologues of *FATTY ACID DESATURASE 2*, particularly *Bna.FAD2.C5*. Our aim was to develop rapeseed lines combining high erucic and low PUFA characters and to assess the impact on thermal stability of the oil they produce. The new type of rapeseed oil (high erucic low polyunsaturate; HELP) contained a substantially greater proportion of erucic acid (54%) compared with high erucic rapeseed oil (46%). Although the total VLCFA content was greater in oil from HELP lines (64%) than from high erucic rapeseed (57%), analysis of triacylglycerol composition showed negligible incorporation of VLCFAs into the sn‐2 position. Rancimat analysis showed that the thermal stability of rapeseed oil was improved greatly as a consequence of reduction of PUFA content, from 3.8 and 4.2 h in conventional low erucic and high erucic rapeseed oils, respectively, to 11.3 and 16.4 h in high oleic low PUFA (HOLP) and HELP oils, respectively. Our results demonstrate that engineering of the lipid biosynthetic pathway of rapeseed, using traditional approaches, enables the production of renewable industrial oils with novel composition and properties.

## Introduction

**Figure 1:**
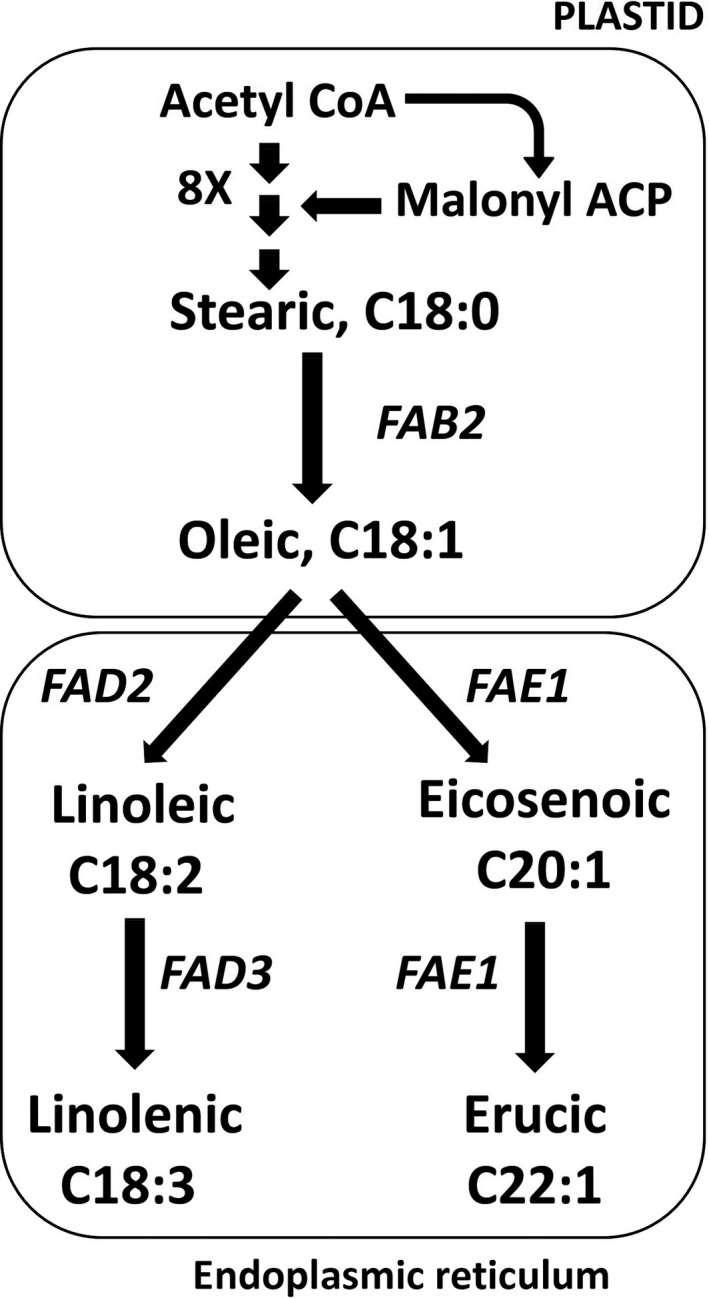
A simplified overview of the fatty acid biosynthesis pathway in *A. thaliana* seeds*.* In the Brassicaceae, the predominant product of fatty acid biosynthesis in the plastid is oleic acid (denoted C18:1 to indicate an acyl chain comprising 18 carbon atoms and containing 1 double bond). Further elaboration of acyl chains is catalysed by microsomal enzymes that either extend the acyl chains, for example to produce the very‐long‐chain fatty acids (VLCFAs) C20:1 and C22:1, or increase the extent of desaturation, that is the number bonds, for example to produce the polyunsaturated fatty acids (PUFAs) C18:2 and C18:3.

Oilseed rape (OSR) is the world’s third largest source of vegetable oil, after palm and soybean (FAO, 2018). It is a crop type of *Brassica napus*, which is a polyploid species (AACC, 2n = 38) formed by the spontaneous hybridization of *B. rapa* (AA, 2n = 20) and *B. oleracea* (CC, 2n = 18) (Nagaharu, 1935; Palmer *et al.*, 1983; Parkin *et al.*, 1995). *Brassica* is the crop genus most closely related to the widely used model plant species *Arabidopsis thaliana* (Yang *et al.*, 1999), in which the lipid biosynthesis pathways leading to the complex suite of triacylglycerol (TAG) species found in storage oil have been extensively characterized (Li‐Beisson *et al.*, 2013). An overview of key components of the pathway that are responsible for fatty acid synthesis and modification is provided in Figure 1. Rapeseed oil is an edible oil, but due to the presence of very‐long‐chain fatty acids (VLCFAs, carbon chain length ≥20), particularly erucic acid, it also has a wide range of industrial applications (Röbbelen, 1991; Zanetti *et al.*, 2012). Feeding studies involving rats raised false concerns about adverse health effects of erucic acid (Beare *et al.*, 1959; Charlton *et al.*, 1975; Thomasson and Boldingh, 1955; de Wildt and Speijers, 1984), resulting in the breeding of low erucic acid rapeseed (LEAR) varieties for human consumption (Stefansson *et al.*, 1961). High erucic acid rapeseed (HEAR) cultivars contain >45% erucic acid in their oil and are used as a ‘green feedstock’ for the oleochemical industry (Knutsen *et al.*, 2016; Meakin, 2007). Erucic acid containing oils have high degree of lubricity and substantivity (ability to cling to the surfaces) than other oils (Piazza and Foglia, 2001). Erucic acid is derived into products such as erucamide, behenyl alcohol, brassylic acid, pelargonic acid, behenic acid, brassinolide and erucyl erucate (Caballero, 2006; Carlson *et al.*, 1977; Molnar, 1974; Neischlag *et al.*, 1967), which are used in the production of a wide range of industrial products, such as lubricants, slip additives, biofuels, pharmaceuticals, cosmetics, jet fuels, plasticizers, soaps, detergents, surfactants, textiles, recording material, rubber, nylon production and many more (Chang *et al.*, 2013; Getachew *et al.*, 2016; Iakovlieva *et al.*, 2017; Johnson and Fritz, 1989; Neischlag *et al.*, 1967; Nieschlag and Wolff, 1971; Pennick *et al.*, 2012; Piazza and Foglia, 2001; Van Dyne and Blasé, 1991; Zanetti *et al.*, 2012). Although other species have been evaluated for the production of erucic acid, the main source remains OSR (Hebard, 2016; Lalas *et al.*, 2012; Sanyal *et al.*, 2015).

Studies in *A. thaliana* have shown the β‐ketoacyl‐CoA synthase encoded at the *FATTY ACID ELONGASE1* (*FAE1*) locus to be rate limiting for the conversion of oleic acid (C18:1) to VLCFAs, and its orthologues have the same function in *B. napus* (Cassagne *et al.*, 1987, 1994; James and Dooner, 1990; James *et al.*, 1995; Kunst *et al*., 1992; Lemieux *et al.*, 1990; Millar and Kunst, 1997; Roscoe *et al.*, 2001; Stumpf and Pollard, 1983; Suneja *et al.*, 1991). The same enzyme is required for two elongation steps: oleic to eicosenoic (C20:1) and eicosenoic to erucic acid (C22:1) (Kondra and Stefansson, 1965). *B. napus* has two orthologues of *FAE1*: *Bna.FAE1.A8* and *Bna.FAE1.C3*. They act in an additive manner, with both being functional in HEAR varieties and nonfunctional in LEAR varieties (Ecke and Breeding, 1995; Harvey and Downey, 1964; Howell *et al*., 1996; Jourdren *et al.*, 1996; Qiu *et al.*, 2006; Thormann *et al.*, 1996). The proportion of VLCFAs that can be accumulated in storage oil in *B. napus* is limited by the poor affinity of its native lysophosphatidic acid acyltransferase (LPAAT) for acyl groups with more than 18 carbon atoms, meaning that they are not incorporated into the sn‐2 position of the glycerol backbone during TAG biosynthesis (Frentzen, 1993; Katavic *et al.*, 2001; Lassner *et al*., 1996). This results in a hypothetical limit of 66.7% VLCFAs. Up to 72% erucic acid has been achieved in *B. napus* by using a LPAAT transgene from *Limnanthes douglasii* to enable incorporation into the sn‐2 position (Nath, 2008; Nath *et al*., 2007).

Studies in *A. thaliana* have shown the oleate desaturase encoded at the *FATTY ACID DESATURASE 2* (*FAD2*) locus to control the conversion of oleic acid (C18:1) to linoleic acid (C18:2) PUFAs (Miquel and Browse, 1992; Okuley *et al.*, 1994). *B. napus* has four orthologues of *FAD2*: *Bna.FAD2.A1, Bna.FAD2.A5*, *Bna.FAD2.C1* and *Bna.FAD2.C5*, corresponding to *BnaA.FAD2.b*, *BnaA.FAD2.a, BnaC.FAD2.b* and *BnaC.FAD2.a*, respectively, in previous studies (Hu *et al.*, 2006; Scheffler *et al.*, 1997; Schierholt *et al*., 2000; Smooker *et al.*, 2011; Yang *et al.*, 2012). A recent study (Wells *et al.*, 2014) showed that, in rapeseed variety Cabriolet, only *Bna.FAD2.C5* is functional. Thus, *Bna.FAD2.C5* was targeted for chemical mutagenesis, resulting in population JBnaCAB_E, from which an allelic series of mutations of this gene was characterized. Several mutant lines produced seeds containing greatly reduced content of PUFAs, which was the aim of the study as polyunsaturation is anticipated to confer thermal instability (Browse *et al.*, 1998; *Durrett et al*., 2008). However, this and a subsequent study (Bai *et al.*, 2019) stopped short of confirming the predicted impact on thermal stability of rapeseed oil. An improvement in thermal stability improves the shelf life of edible oils (Kinney and Clemente, 2005) but would be transformative for industrial applications where greater thermal stability is required, such as feedstock for lubricants and hydraulic fluids, whilst minimizing environmental impact by retaining biodegradability (which mineral oil lacks).

In this study, we aimed primarily to characterize the impact on seed oil fatty acid composition of combining the low PUFA trait with the high erucic trait and thereby improving our understanding of the factors that limit erucic acid content. Having developed such rapeseed lines, we could address our secondary aim of assessing the impact of reducing PUFAs on the thermal stability of oil. Importantly, we used a strategy for development of the material that used genomics to accelerate ‘traditional’ mutation breeding, resulting in cultivars that are not considered genetically modified (GM) organisms.

## Results

### HELP development

We combined low PUFA and high erucic acid seed oil traits by crossing the low PUFA donors K0472 and K0047 (which had been generated by chemical mutagenesis of the low erucic acid variety Cabriolet as reported by Wells *et al.* (2014)) onto the current high erucic acid rapeseed cultivar Maplus. In F_1_ progeny (successful crosses assessed by *Bna.FAD2* genotyping), two lines were randomly selected from each cross‐combination, grown and self‐pollinated to produce F_2_ progeny. Marker‐assisted selection was used in F_2_ generation to identify plants inheriting appropriate alleles at the two loci in this cross conferring the low PUFA trait (the inactive allele *Bna.fad2.A5* and mutated versions of *Bna.FAD2.C5* denoted either *Bna.fad2.C5* ^K0472^ or *Bna.fad2.C5* ^K0047^, depending on donor) along with the two alleles conferring the high erucic acid trait (*Bna.FAE1.A8* and *Bna.FAE1.C3*). PCR primers and allele sequences are shown in Table S1, and sequence alignments of the amplicons of F_2_ plants are shown in Appendix [Link], [Link], [Link]. We had been unable to amplify the third orthologue of *FAD2* reported previously as being required in inactive form for manifestation of the low PUFA trait (*Bna.fad2.C1*), and all individuals selected as inheriting low PUFA alleles *Bna.fad2.A5* and *Bna.fad2.C5* had inherited the low PUFA trait. From this, we deduce that variety Maplus already has the inactive (deleted) allele *Bna.fad2.C1* as well as the inactive allele *Bna.fad2.A1* that appears common in European rapeseed (Wells *et al.*, 2014). Out of 192 F_2_ plants, one HELP plant ‘2‐91’ from cross Maplus x K0472 (K0472‐HE) and another HELP plant ‘4‐87’ from cross Maplus x K0047 (K0047‐HE) were selected. These were self‐pollinated up to F_5_ progeny, and the seeds were collected for fatty acid analysis. The summary of the development of HELP lines is shown in Figure S1.

### Seed fatty acid composition of HELP lines

Fatty acid profile was analysed from F_3_ seeds of two HELP lines, 2‐91 (K0472‐HE) and 4‐87 (K0047‐HE), and high erucic acid control, Maplus as shown in Table 1. In comparison with Maplus, both HELP lines showed a slight increase in the very‐long‐chain fatty acids (sum of C20:1, C22:1 and C24:1) but showed a large decrease in the polyunsaturates (C18:2 and C18:3) percentages. As there were no biological replicates available for both HELP lines, F_3_ progeny was multiplied to F_4_ generation to assess the stability of fatty acid composition.

**Table 1:** Seed fatty acid composition (% by weight, mean ± SD) of HELP F_3_ progeny and HEAR control, Maplus

Line	Palmitic C16:0	Palmitoleic C16:1	Stearic C18:0	Oleic C18:1	Linoleic C18:2	Linolenic C18:3	Arachidic C20:0	Eicosenoic C20:1	Behenic C22:0	Erucic C22:1	Lignoceric C24:0	Nervonic C24:1
K0472‐HE (2‐91)	2.2 ± 0.1	0.3 ± 0.1	0.7 ± 0.0	27.1 ± 0.5	1.7 ± 0.0	3.2 ± 0.1	0.6 ± 0.0	8.4 ± 0.1	0.4 ± 0.0	53.7 ± 1.0	0.1 ± 0.1	1.4 ± 0.9
K0047‐HE (4‐87)	2.3 ± 0.1	0.2 ± 0.0	0.7 ± 0.0	25.2 ± 0.1	2.9 ± 0.1	4.8 ± 0.0	0.6 ± 0.0	9.1 ± 0.0	0.4 ± 0.0	52.6 ± 0.5	0.1 ± 0.0	0.9 ± 0.3
Maplus	3.6 ± 0.1	0.2 ± 0.1	0.8 ± 0.0	12.3 ± 0.3	12.4 ± 0.0	8.2 ± 0.0	0.7 ± 0.0	8.7 ± 0.1	0.6 ± 0.0	51.1 ± 0.6	0.1 ± 0.0	0.8 ± 0.2

Each fatty acid value is the mean of three technical replicates.

**Figure 2:**
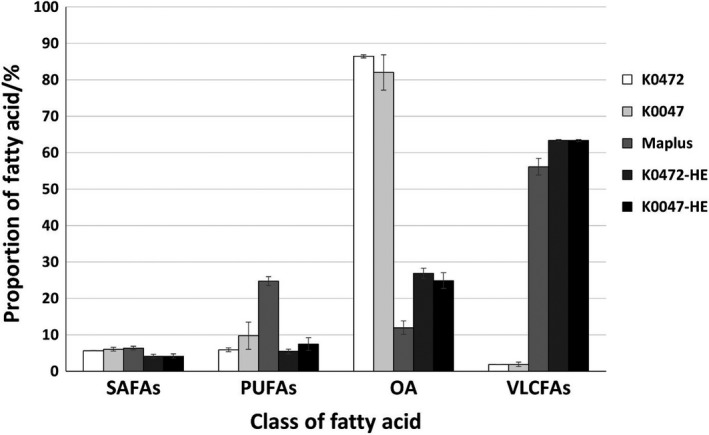
Seed fatty acid composition. Composition classified by fatty acid type: saturated fatty acids (SAFAs) = sum of C16:0, C18:0, C20:0, C22:0 and C24:0; polyunsaturated fatty acids (PUFAs) = sum of C18:2 and C18:3; oleic acid = C18:1; very‐long‐chain fatty acids (VLCFAs) = sum of C20:1, C22:1 and C24:1. Mean values plotted for 10 biological replicates each of K0472, K0047, Maplus, K0472‐HE and K0047‐HE, respectively. Standard deviation is shown as error bars.

Fatty acids were analysed for F_4_ seeds from 10 individual plants from each of the crosses, Maplus x K0472 and Maplus x K0047 (designated HELP lines K0472‐HE and K0047‐HE, respectively), along with parental lines as controls. The results are summarized in Table 2, and detailed fatty acid profiles for individual plants are provided in Appendix [Link], [Link], [Link]. Compared with Maplus, both HELP lines showed large increases in content of the main VLCFAs eicosenoic (C20:1), erucic (C22:1) and nervonic acid (C24:1) as shown in Figure 2. These increased from 55.7% of total fatty acids in Maplus to 63.3% in each of K0472‐HE and K0047‐HE HELP lines. Erucic acid increased from 46.1% in Maplus to 53.7% in K0047‐HE and 54.4% in K0472‐HE. One‐way ANOVA (analysis of variance) was calculated on five genotypes (having 10 replicates each)—Maplus, K0472, K0047, K0472‐HE and K0047‐HE. The *P*‐values for each of VLCFAs, PUFAs, OA and SAFAs were found to be <2 × 10^−^^16^ (highly significant), and thus, these fatty acid groups differ significantly from each other. To know the difference in the individual genotypes, post hoc Tukey’s test was performed. The *P*‐values for the genotype groups, Maplus‐K0047 and Maplus‐K0472 were found to be highly significant. Thus, HELP lines differ significantly from HEAR, Maplus for these fatty acid groups. The relative proportions of the two most abundant VLCFAs (C20:1 and C22:1) varied between the individual plants representing the HELP lines and showed a strong inverse correlation (*R* ^2^ = 0.795), as shown in Figure S2. It shows that in some instances, the elongation from oleic acid (C18:1) goes up to eicosenoic acid and is not elongated to erucic acid, but the overall VLCFA composition remains constant.

**Table 2:** Seed fatty acid composition (% by weight, mean ± SD) of HELP F_4_ progeny and parental controls

Line	Mean fatty acid percentage composition of seed ± standard deviation											
	Palmitic C16:0	Palmitoleic C16:1	Stearic C18:0	Oleic C18:1	Linoleic C18:2	Linolenic C18:3	Arachidic C20:0	Eicosenoic C20:1	Behenic C22:0	Erucic C22:1	Lignoceric C24:0	Nervonic C24:1
K0472	3.4 ± 0.1	0.2 ± 0.2	1.2 ± 0.3	86.4 ± 1.4	1.8 ± 0.3	4.0 ± 0.5	0.6 ± 0.2	1.8 ± 0.2	0.2 ± 0.2	0.0 ± 0.1	0.2 ± 0.2	0.0 ± 0.1
K0047	4.0 ± 0.3	0.2 ± 0.1	1.1 ± 0.2	82.0 ± 2.1	3.2 ± 0.6	6.5 ± 1.2	0.4 ± 0.2	1.8 ± 0.2	0.3 ± 0.2	0.0 ± 0.1	0.2 ± 0.1	0.1 ± 0.0
Maplus	4.2 ± 0.5	0.3 ± 0.1	0.8 ± 0.1	12.2 ± 1.8	16 ± 1.3	8.9 ± 1.0	0.7 ± 0.1	8.8 ± 1.3	0.6 ± 0.0	46.1 ± 2.3	0.2 ± 0.1	0.8 ± 0.2
K0472‐HE (2‐91‐1 to 10)	2.5 ± 0.4	0.2 ± 0.1	0.7 ± 0.1	26.8 ± 1.6	1.8 ± 0.4	3.6 ± 0.6	0.6 ± 0.1	8.1 ± 1.6	0.3 ± 0.1	54.4 ± 3.0	0.0 ± 0.0	0.8 ± 0.1
K0047‐HE (4‐87‐1 to 10)	2.2 ± 0.1	0.2 ± 0.0	0.8 ± 0.1	24.9 ± 1.3	2.7 ± 0.5	4.7 ± 0.5	0.7 ± 0.0	8.8 ± 1.2	0.4 ± 0.0	53.7 ± 1.7	0.1 ± 0.1	0.8 ± 0.0

Each fatty acid value is the mean of 10 biological replicates.

In addition to the anticipated increase in the content of VLCFAs, the introduction of the low PUFA trait affected the content of other fatty acids as shown in Figure 2. As expected, compared with Maplus, both HELP lines showed large decreases in content of the main PUFA linoleic (C18:2) and linolenic acid (C18:3). These decreased from 24.9% of total fatty acids in Maplus to 5.4% in K0472‐HE and 7.4% in K0047‐HE. The proportion of saturated fatty acids (SAFAs) also reduced, with the total of all SAFAs reducing from 6.5% in Maplus to 4.1% in K0472‐HE and 4.2% in K0047‐HE. Both PUFAs and SAFAs are lower in the HELP lines than they are in the low erucic acid donors of the PUFA trait (6.6% and 7.0% SAFAs in K0472 and K0047, respectively; 7.0% and 8.9% PUFAs in K0472 and K0047, respectively).

In order to assess the stability of the HELP profile in further generations, 169 F_4_ plants were grown on, and selfed seed (F_5_ generation) was collected and analysed for fatty acid composition. The detailed fatty acid profiles for individual plants are provided in Appendix [Link], [Link], [Link]. These showed that the HELP profile is stably inherited. The relative proportions of erucic and eicosenoic acid were analysed. As illustrated in Figure S3, this showed correlation between the F_5_ and F_4_ generations. This suggests a heritable basis for the variation, such as a minor effect locus (the same alleles are present for the *FAE1* orthologues, so the difference is not attributable to allelic variation in these).

### Pattern of very‐long‐chain fatty acid incorporation into triacylglycerol

The increased proportion of VLCFAs in the HELP lines (63.3%) approaches the hypothetical limit for TAG in *Brassica* species (66.7%) imposed by the inability of Brassica LPAAT to incorporate such moieties at the sn‐2 position of the TAG molecule. We therefore tested whether, given the large proportion of VLCFAs that are synthesized and accumulated, incorporation had taken place at this position. Total lipids were extracted from HELP (K0472‐HE, F_4_ seeds) and its progenitors (Maplus and K0472) and TAGs analysed for fatty acids incorporated at the sn‐2 position (van Erp *et al.*, 2010). The results, illustrated in Table S2, show negligible incorporation at the sn‐2 position, meaning that VLCFA occupation at the sn‐1 and sn‐3 positions can be calculated as ~95% (i.e. 63.3% observed of the 66.7% available).

### Thermal stability of low polyunsaturated rapeseed oils

The reduced PUFA content of HELP rapeseed oil is predicted to improve thermal stability. To test this, we produced oil from field‐grown low PUFA lines K0472‐HE and K0472. The crude oil was subjected to Rancimat stability testing (Läubli *et al*., 1988), along with field‐grown conventional high and low erucic rapeseed oils (HEAR variety Maplus and LEAR variety Nikita, respectively). The fatty acid compositions of the crude oils are shown in Table S3, and the results of the stability test are shown in Table 3. As predicted, thermal stability improved greatly with the reduction in PUFA content. The stability of low erucic oil (variety Nikita) improved from 3.8 h for conventional LEAR rapeseed oil to 11.3 h for HOLP rapeseed oil. The stability of high erucic oil (variety Maplus) improved from 4.2 h for conventional HEAR to 16.4 h for HELP. The oxidative capacity of the oil types was assessed (shown in Table S4) and was not correlated with thermal stability.

**Table 3:** Rancimat analysis of oil thermal stability

Oil type	Source	Induction time at 120 °C (h)† Mean ± SD
Low erucic acid rapeseed (LEAR)	LEAR (variety Nikita)	3.8 ± 0.08
High erucic acid rapeseed (HEAR)	HEAR (variety Maplus)	4.2 ± 0.03
High oleic low polyunsaturated (HOLP)	HOLP (line K0472)	11.3 ± 0.17
High erucic low polyunsaturated HELP)	HELP (line K0472‐HE)	16.4 ± 0.24

^†^ Each value is mean of four technical replicates.

## Discussion

Despite the opportunities for exploiting high erucic rapeseed oil as a renewable feedstock, growing demand from the oleochemical industry and its widespread cultivation, there has been relatively little progress increasing the proportion of erucic acid in oil produced commercially (Iakovlieva *et al.*, 2017; Johnson and Fritz, 1989; Knutsen *et al.*, 2016; Meakin, 2007; Nieschlag and Wolff, 1971; Röbbelen, 1991; Zanetti *et al.*, 2012). Transgenic approaches have enabled exploration of one limitation in the accumulation of erucic acid in storage lipid, that imposed by the *Brassica* LPAAT enzyme, which cannot incorporate VLCFAs at the sn‐2 position of TAG (Bernerth and Frentzen, 1990; Brockerhoff, 1971; Cao *et al*., 1990; Frentzen, 1993; Katavic *et al.*, 2001; Nath, 2008; Nath *et al*., 2007; Nath *et al.*, 2009; Sasongko and Möllers, 2005). The analysis we undertook of FA composition at TAG sn‐2 positions (Table S2) confirmed expectations that even in the presence of additional substrate, VLCFAs are not incorporated at this position. Our studies do, however, provides insights into limitations imposed by the availability of monounsaturated 18‐carbon moieties for elongation to VLCFAs. Recapitulation of the combination of *Bna.fad2* mutations shown previously to greatly restrict the conversion of oleic acid to PUFAs (Wells *et al.*, 2014), but, in a high erucic acid background, increases the availability of C18:1 for extension by the *Brassica* elongases (controlled by the *Bna.FAE1* loci). The observed impact is to increase the proportion of erucic acid in seed oil (by ~17% of its original value), demonstrating that the availability of C18:1 is, indeed, an important limitation for erucic acid content. To increase erucic acid content further, the introduction of an LPAAT capable of incorporation of VLCFAs at the sn‐2 position would be required. However, the pull through to VLCFAs already observed decreases the content of both saturated and polyunsaturated fatty acids (by ~38% and ~23%, respectively, of their values in the low PUFA donor line K0472), resulting in the oil produced by K0472‐HE containing ~90% monounsaturated fatty acids.

The same enzyme complex extends oleic (C18:1) to eicosenoic (C20:1) and eicosenoic to erucic acid (C22:1) (Kondra and Stefansson, 1965; Lassner *et al*., 1996). We observed variability in the content of eicosenoic, along with a negative correlation between contents of eicosenoic and erucic acids (Figure S2). Similar observations have been made previously in the progeny produced by crossing high erucic acid rapeseed with high oleic acid rapeseed (Sasongko and Möllers, 2005). It has been suggested that different alleles of *B. napus* orthologues of *FAE1* have a different potential of producing erucic acid (Mahmood *et al.*, 2003). This would not be unprecedented as, for example, the elongase encoded by the *A. thaliana FAE1* locus uses oleic as a preferred substrate, resulting in the accumulation of eicosenoic rather than erucic acid (Katavic *et al.*, 2001). In contrast, our results are consistent with the hypothesis that elongase activity is limiting the content of erucic acid in low PUFA lines, K0472‐HE and K0047‐HE. The observation of heritability to the F_5_ generation, as shown by correlation of the relative proportions of erucic and eicosenoic in F_4_ individuals and their progeny, indicates a genetic basis. As there is no variability for the active alleles of *Bna.FAE1* in our experiments (these being inherited from Maplus), our observations can be attributed to the presence of ‘modifier’ loci for erucic acid content. An associative transcriptomics analysis of seed erucic acid content in a genetic diversity panel of *B. napus* indicated such a locus may be present on chromosome A5, with a gene encoding a fatty acid hydroxylase 1 identified as a candidate (Havlickova *et al.*, 2018).

An increased content of erucic acid in HELP oil would undoubtedly be beneficial for industries producing compounds such as erucamide, for which rapeseed is the major source of erucic acid (Zanetti *et al.*, 2012). HELP lines are expected to be superior to existing HEAR varieties because they contain a higher proportion of erucic acid in their oil (i.e. the yield of the primary product is greater) and due to anticipated reduction in processing costs. There is also an increasing global demand for more ‘environmental‐friendly’ alternatives to mineral oil. The key drawback of rapeseed oil (and, indeed for vegetable oils in general) is the thermal instability that is a consequence of their high content of PUFAs. To test our prediction that low PUFA rapeseed oil (both HOLP and HELP types) would have substantially greater thermal stability than conventional rapeseed oil, we tested this using an industry standard test (Rancimat; Läubli *et al*., 1988). The results confirmed a dramatic improvement compared with conventional rapeseed oil. For HOLP, the oil is suitable for edible uses such as repeated high‐temperature frying and retains the benefit of low saturate content. For HELP, the oil is a ‘non‐GM green feedstock’ for the production of large volumes of low‐cost, biodegradable products such as lubricants and hydraulic fluids, enabling more widespread use in sensitive environments. The development of non‐GM varieties even for industrial crops is important as the cost of deregulation (a requirement due to the release of the organisms into the environment) is prohibitive and many countries do not permit the cultivation of GM crops at all.

## Experimental procedures

### Plant material and growth conditions

Donors of the low PUFA trait were selected from the ethyl methane sulphonate‐mutagenized population JBnaCAB_E, derived from the winter oilseed rape variety Cabriolet (Wells *et al.*, 2014). These lines carried mutations in *Bna.FAD2.C5*: K0472 and K0047. The donor of the high erucic acid trait was the current, open‐pollinated high erucic acid winter rapeseed variety Maplus (breeder: NPZ‐Lembke, Germany; https://www.npz.de/).

Seeds were sown in the medium‐grade compost (Scotts Levington F2 + S) and kept in the glasshouse under long‐day conditions of 16‐h photoperiod and temperatures of 20 °C/14 °C for day/night. At four‐leaf stage (after ~3 weeks of sowing), the plants were vernalized for 6 weeks with 8‐h photoperiod at 4 °C (vernalization being necessary as all lines are winter habit).

### Development of HELP lines

K0472 and K0047 were crossed, as pollen donor, onto Maplus. F_1_ progeny were genotyped by PCR amplification and sequencing, with primers and alleles as indicated in Supplementary Table 1, to confirm the crosses. Two lines were randomly selected from each cross‐combination, grown and self‐pollinated. Ninety‐six F_2_ plants were grown for each and analysed for alleles of *Bna.FAD2.A5, Bna.FAD2.C5, Bna.FAE1.A8* and *Bna.FAE1.C3* by PCR amplification and sequencing, with primers and alleles as indicated in Table S1. Trace files were analysed using software Mutation Surveyor® (SoftGenetics LLC, State College, PA). One HELP plant ‘2‐91’ from cross Maplus x K0472 (K0472‐HE) and another HELP plant ‘4‐87’ from cross Maplus x K0047 (K0047‐HE) were confirmed to have inherited the required combination of homozygous alleles: *Bna.FAE1.A8, Bna.FAE1.C3, Bna.fad2.A5* and *Bna.fad2.C5*. The sequence alignment files are provided in Appendix [Link], [Link], [Link]. The plants were self‐pollinated and the lines amplified to F_4_ for analysis.

### DNA extraction and PCR amplification

One young leaf per plant was sampled at three‐leaf stage, and ‘DNeasy Plant 96 Qiagen Kit for 96 samples’ was used for the automated isolation of the DNA according to the manufacturer’s instructions (Qiagen, UK). The details of the primer pairs used for the amplification of *Bna.FAD2.C5*, *Bna.FAD2.A5*, *Bna.FAE1.A8* and *Bna.FAE1.C3* copies are shown in Table S1. DNA amplification was carried out in volumes of 25 μl with 50 ng of gDNA, 0.4 μM each of forward and reverse primers and 1× master mix (Thermo Scientific, Wilmington, DE). For *Bna.FAD2* orthologues amplification, the PCR profile used was as follows: initial denaturation of 94 °C for 5 min; 35 cycles each with 94 °C for 30 s, 57 °C for 30 s and 72 °C for 1 min; final extension of 72 °C for 10 min. For *Bna.FAE1* orthologues, the touchdown PCR profile was used: initial denaturation of 94 °C for 5 min; 15 cycles each with 94 °C for 30 s, 63 °C for 30 s (decrease by 1 °C every cycle) and 72 °C for 1 min; and 30 cycles each with 94 °C for 30 s, 53 °C for 30 s and 72 °C for 1 min; and final extension of 72 °C for 15 min. 1.5% agarose gel was used at 100 volts for 30 min for the separation of the PCR products, and these were observed under UV gel viewer.

### PCR product purification, sequencing and analysis

PCR products were purified using the ‘Mag‐Bind® RXNPurePlus’ by following manufacturer’s protocol. Genewiz (https://www.genewiz.com) was used for the Sanger sequencing of the purified PCR products. Mutation surveyor® v5.0 software (SoftGenetics LLC, State College, PA) was used for the detection of the mutations from trace files.

### Fatty acid composition analysis

Four milligrams of tripentadecanoin (Sigma‐Aldrich, Poole, Dorset, UK) internal standard was added to 30 mg of seeds, followed by the addition of 400 µL of cold hexane: isopropanol (3:2 v/v) solution. The seeds were ground using a Tissuelyser and incubated for 1 h on ice. The samples were centrifuged at ~9600 ***g*** for 5 min, and supernatant was transferred to a new tube. The pellet was washed another two times with 400 µL of cold hexane: isopropanol solution, vortexed, centrifuged and supernatants were pooled to the same tube. Then, 600 µL of 6.7% Na_2_SO_4_ was added, vortexed and centrifuged for 30 s. The upper phase was removed into a new tube and dried in the Genevac^TM^ (EZ‐2.3 Elite model). These were reconstituted in 750 µL chloroform. 1/15^th^ part of the sample (2–3 technical replicates) was transferred to a 2‐mL screw cap Supelco® glass vial. It was followed by the addition of 750 µL hexane and 500 µL 1N methanolic hydrochloric acid; incubation at 85 °C for 3 h; and addition of 250 µL of 0.9% KCl. The vials were vortexed, and upper hexane layer containing fatty acid methyl esters (FAMEs) was used the gas chromatography (GC) analysis. FAMEs were analysed on Thermo Scientific’s Trace GC Ultra‐FID with GC column (specifications: BP × forte 10 m × 0.1 mm ID × 0.2 m film thicknesses) with a run time of ~5 min per sample. Supelco® 37 component FAME mix (Sigma‐Aldrich) was used as an external standard, and hexane was used as a blank. Data were analysed with the Thermo Scientific’s ChromQuest^TM^ software (version 4.2.34) platform.

### Lipid extraction for TAG analysis

One millilitre of isopropanol containing butylated hydroxy toluene (BHT) was added to 4–6 seeds of each sample and heated in the oven at 85 °C for 15 min. Heat‐quenched samples were homogenized in a homogenizer; then, 2 mL of chloroform and 3 mL of methanol were added to each sample. It was followed by the addition of 1.6 mL of water, 2 mL of chloroform and 2 mL of 0.88% (w/v) KCl and gentle mixing. The tubes were centrifuged for 2 min at 400 ***g***. The bottom phase was collected carefully in another tube and dried under N_2_. The lipid was resuspended in 500 µL of toluene containing 0.005% BHT for further analysis. FAMEs were derived using 10 µL of this sample, and total lipid content was calculated using GC results.

### Positional distribution analysis of TAGs

Total lipid (1500 µL) was loaded on the thin‐layer chromatography (TLC) plate (20 × 20 cm, silica), and the mixture was separated using hexane: diethylether: acetic acid (70:30:1, v/v). TAGs were eluted from TLC silica twice by washing with 5 mL of chloroform: methanol (4:1, v/v). A phase separation was induced by adding 2 mL of methanol and 4 mL of 0.88% (w/v) potassium chloride. The chloroform phase was collected, and the aqueous phase was back extracted with 5 mL of chloroform. The chloroform phase was dried under N_2_ and re‐suspended in 500 L of toluene containing 0.005% BHT. TAGs were derivatized to FAMEs by using 10 µ of this sample and analysed by GC. Samples were dried down using N_2_, and 1 mg of TAG was resuspended in: 1 mL of diethylether, 0.8 mL of buffer (50 mM NaBr, 5 mM CaCl_2_, pH 7.6) and 200 µ of lipase (*Rhizomucor miehei* lipase; Sigma‐Aldrich). It was vortexed for 40 min, and the reaction was stopped by the addition of 2 mL of methanol: chloroform (1:1, v/v). The chloroform layer was collected, dried down and resuspended in 200 µ of chloroform. The mixture was separated by TLC using hexane: diethyl ether: acetic acid (35:70:1.5, v/v). 2‐monoacylglycerols (2‐MAGs) and TAGs were scrapped from the plate, and FAMEs were derived and analysed by GC.

### Oxidative stability analysis

HOLP and HELP plants were grown in Moravian‐Silesian region of Czech Republic (49°56′N, 17°54′E), sown September 2017, harvested July 2018. Conventional low and high erucic rapeseed was sourced from the breeders (location grown not specified). Oil pressing was carried out using a small capacity Komet screw press (Model CA 59 G; IBG Monforts, Mönchengladbach, Germany), with a 6‐mm press nozzle die and a screw speed of 20 rpm. The oxidative stability of the pressed oils was determined using a ‘Metrohm Rancimat model 743’, according to AOCS Official Method Cd 12b‐92. Briefly, the induction times (*n* = 4) for portions of oil (3.0 g) was determined at 120 °C with 10 L/h air throughput. The induction point, as a measure of the oil stability index (OSI), is the time point of maximal change in the rate of oxidation.

### Antioxidant properties of oil samples

The total antioxidant capacity (TAC) of oils was determined using a commercial kit (Sigma‐Aldrich, Catalogue number MAK187) to measure the concentration of the small molecule and protein antioxidants. This method is based on the detection of reduced Cu^+^ ion chelates with colorimetric probe at absorbance 570 nm, which is proportional to the total antioxidant capacity in Trolox equivalents (a water‐soluble vitamin E analogue used as an antioxidant standard). The absorbance at 570 nm was measured using spectrophotometric plate reader (Clariostar, BMG LABTECH Ltd. Oterberg, Germany). The concentrations of the samples were then calculated with the value obtained from trolox standards.

## Conflict of interests

The authors declare no competing financial interests.

## Author contributions

H.K., P.E. and I.B. conceived and designed the project. H.K., L.W., N.S., R.S and H.v.E. performed the experiments and analysed the results. H.K. and I.B wrote the manuscript. N.S., R.S. and P.E. revised the manuscript.

## Supporting information


**Figure S1** Summary of the development of HELP lines, K0472‐HE (2‐91) and K0047‐HE (4‐87).

**Figure S2** Relationship between erucic and eicosenoic acid content of seeds of K0472‐HE and K0047‐HE.

**Figure S3** Relationship between erucic / eicosenoic acid ratios in F4 plants and their F5 progeny.

**Table S1** PCR primers used for selecting alleles required in HELP lines.

**Table S2** Fatty acid composition at TAG sn‐2 position.

**Table S3** Fatty acid composition of oils subjected to thermal stability testing.

**Table S4** Total antioxidant capacity of oils subjected to thermal stability testing.

**Appendix S1** Sequence alignment of amplicons of *Bna.FAE1.A8, Bna.FAE1.C3, Bna.FAD2.A5 *and *Bna.FAD2.C5 *copies.

**Appendix S2** Detailed fatty acid composition of HELP F_4_ seeds. For each sample, the fatty acid percentage represents the mean of three technical replicates.

**Appendix S3** Detailed fatty acid composition of HELP F_5_ seeds.
