# Supplementary figures and images for: The impact of reducing fatty acid desaturation on the composition and thermal stability of rapeseed oil
Source: Plant Biotechnol J. 2019 Oct 14;18(4):983–91. doi: 10.1111/pbi.13263 (PMC7061866; doi:10.1111/pbi.13263)

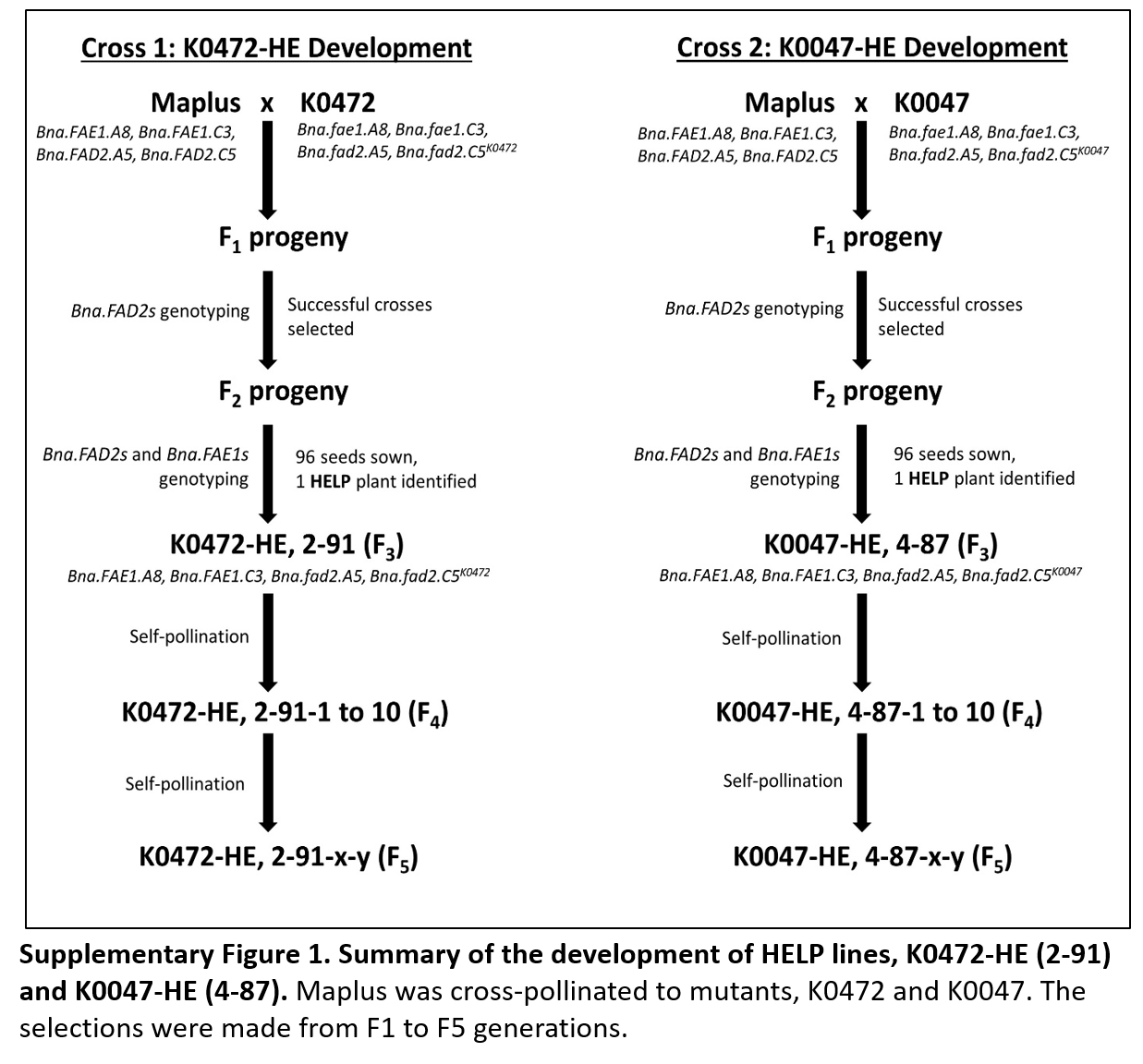

Supplement: Supplementary file 1 — Figure S1 Summary of the development of HELP lines, K0472‐HE (2‐91) and K0047‐HE (4‐87). [file PBI-18-983-s010.tif]

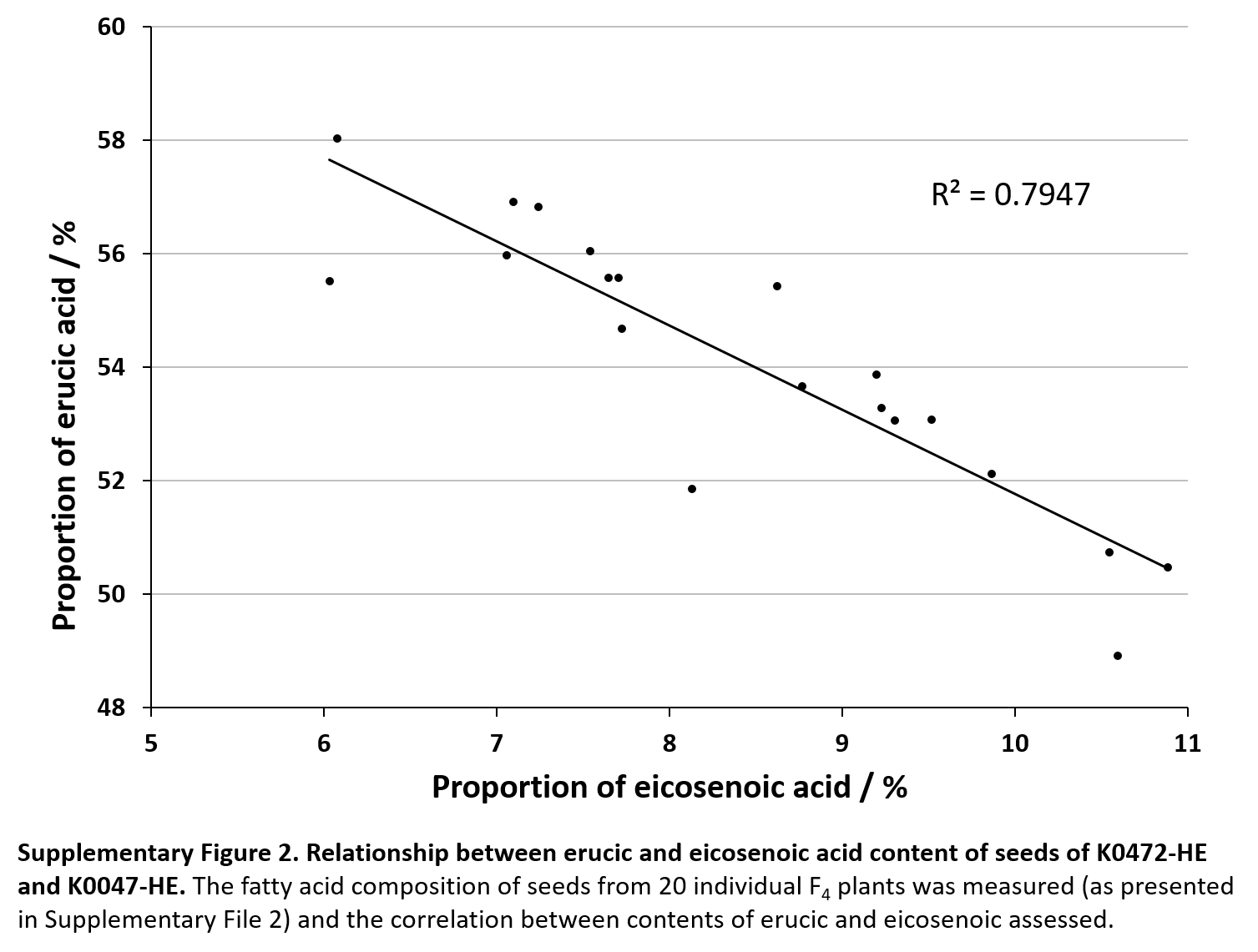

Supplement: Supplementary file 2 — Figure S2 Relationship between erucic and eicosenoic acid content of seeds of K0472‐HE and K0047‐HE. [file PBI-18-983-s009.tif]

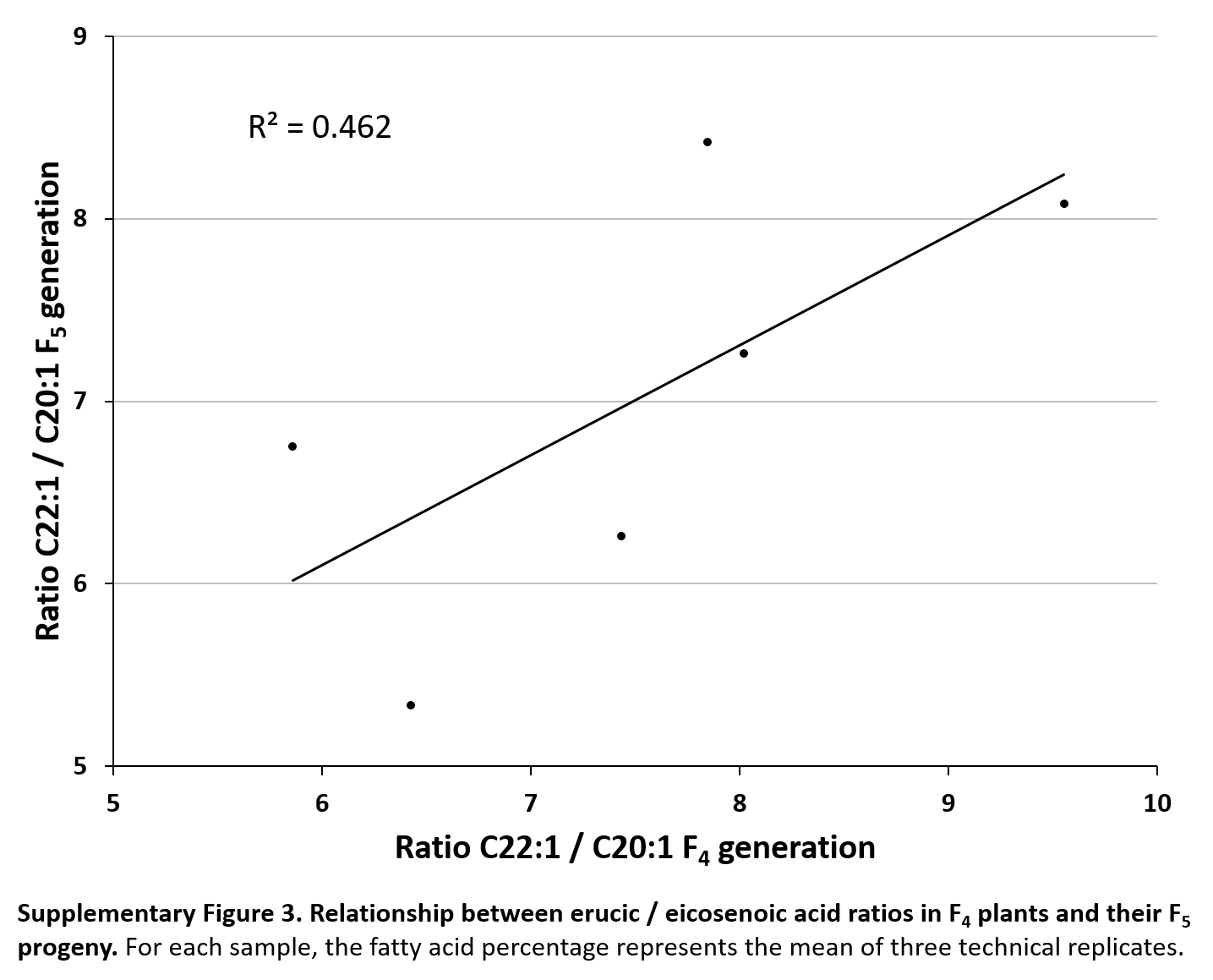

Supplement: Supplementary file 3 — Figure S3 Relationship between erucic / eicosenoic acid ratios in F4 plants and their F5 progeny. [file PBI-18-983-s008.tif]
