# Supplementary material for: The impact of reducing fatty acid desaturation on the composition and thermal stability of rapeseed oil
Source: Plant Biotechnol J. 2019 Oct 14;18(4):983–91. doi: 10.1111/pbi.13263 (PMC7061866; doi:10.1111/pbi.13263)
Supplement: Supplementary file 4 — Table S1 PCR primers used for selecting alleles required in HELP lines. [file PBI-18-983-s007.docx]

Supplementary Table 1. PCR primers used for selecting alleles required in HELP lines

| **Locus** | **Forward primer (5’ – 3’)** | **Reverse primer (5’ – 3’)** | **Amplicon Length (bp)** | **Functional allele** | **Non-functional allele** | **Position in K0472 (coordinate)** | **Position in K0047 (coordinate)** |
| --- | --- | --- | --- | --- | --- | --- | --- |
| *Bna.FAD2.C5* | GTCTCCTCCCTCCAAAAAGT | CAAGACGACCAGAGACAGC | 1212 | G | A | 224 | 656 |
| *Bna.FAD2.A5* | GTGTCTCCTCCCTCCAAA | CCTCATAACTTATTGTTGTACCAG | 1133 | C | Deleted C | 157 | 157 |
| *Bna.FAE1.A8* | TACTCATGCTACCTTCCAC | CCTCTACATCGATCGGTGCT | 1407 | G | A | 823 | 823 |
| *Bna.FAE1.C3* | GCCGCTATTTTGCTCTCCAA | CCAATCAATTCGGGAGCCAC | 922 | TT | Deleted TT | 296, 297 | 296, 297 |
