# Supplementary material for: The impact of reducing fatty acid desaturation on the composition and thermal stability of rapeseed oil
Source: Plant Biotechnol J. 2019 Oct 14;18(4):983–91. doi: 10.1111/pbi.13263 (PMC7061866; doi:10.1111/pbi.13263)
Supplement: Supplementary file 5 — Table S2 Fatty acid composition at TAG sn‐2 position. [file PBI-18-983-s006.docx]

**Supplementary Table 2. Fatty acid composition at TAG sn-2 position, % by weight.**

| **Line** | **C16:0** | **C18:0** | **C18:1** | **C18:2** | **C18:3** | **C20:0** | **C20:1** | **C20:2** | **C22:0** | **C22:1** | **C24:1** |
| --- | --- | --- | --- | --- | --- | --- | --- | --- | --- | --- | --- |
| K0472-HE | 1.1 ± 0.4 | 1.0 ± 0.4 | 78.9 ± 2.8 | 5.1 ± 0.8 | 10.8 ± 1.3 | <0.1 | 0.9 ± <0.1 | 1.1 ± 0.6 | <0.1 | 0.5 ± 0.1 | <0.1 |
| Maplus | 0.7 ± 0.1 | 0.6 ± 0.1 | 28.5 ± 2.8 | 46.7 ± 1.1 | 21.3 ± 2.0 | <0.1 | 0.3 ± 0 | 0.6 ± 0.1 | <0.1 | 0.17 ± <0.1 | <0.1 |
| K0472 | 0.9 ± 0.3 | 0.7 ± 0.2 | 86.8 ± 0.3 | 3.5 ± 0.1 | 7.4 ± 0.5 | <0.1 | 0 | 0.7 ± 0.2 | <0.1 | 0 | <0.1 |

For each genotype the fatty acid percentage represents the mean ± SE of either three or four biological replicates.
