# Supplementary material for: The impact of reducing fatty acid desaturation on the composition and thermal stability of rapeseed oil
Source: Plant Biotechnol J. 2019 Oct 14;18(4):983–91. doi: 10.1111/pbi.13263 (PMC7061866; doi:10.1111/pbi.13263)
Supplement: Supplementary file 6 — Table S3 Fatty acid composition of oils subjected to thermal stability testing. [file PBI-18-983-s005.docx]

**Supplementary Table 3. Fatty acid composition of oils subjected to thermal stability testing, % by weight**

| **Line** | **C16:0** | **C16:1** | **C18:0** | **C18:1** | **C18:2** | **C18:3** | **C20:0** | **C20:1** | **C22:0** | **C22:1** | **C24:0** | **C24:1** |
| --- | --- | --- | --- | --- | --- | --- | --- | --- | --- | --- | --- | --- |
| Nikita | 4.43 ± 0.10 | 0.24 ± 0.01 | 1.64 ±0.05 | 66.90 ± 0.47 | 16.65 ± 0.23 | 8.20 ± 0.10 | 0.51 ± <0.01 | 1.09 ± 0.01 | 0.25 ± 0.01 | 0.02 ± 0.02 | 0.08 ± <0.01 | 0.00 ± <0.01 |
| Maplus | 3.45 ± 0.07 | 0.24 ± 0.01 | 0.99 ± 0.09 | 14.54 ± 0.32 | 12.79 ± 0.43 | 9.03 ± 0.31 | 0.63 ± 0.05 | 6.04 ± 0.34 | 0.54 ± 0.04 | 51.56 ± 1.40 | 0.20 ± 0.04 | 0.00 ± <0.01 |
| K0472 | 3.12 ± 0.07 | 0.21 ± 0.04 | 1.38 ± 0.05 | 88.00 ± 0.31 | 1.61 ± 0.15 | 3.18 ± 0.24 | 0.39 ± 0.08 | 1.77 ± 0.07 | 0.21 ± 0.06 | 0.09 ± 0.03 | 0.03 ± 0.03 | 0.01 ± 0.01 |
| K0472-HE | 1.85 ± 0.10 | 0.18 ± 0.01 | 0.65 ± 0.06 | 26.73 ± 0.90 | 1.53 ± 0.10 | 3.14 ± 0.16 | 1.24 ± 0.32 | 4.10 ± 0.11 | 0.62 ± 0.03 | 58.81 ± 0.55 | 1.06 ± 0.09 | 0.09 ± 0.13 |

For each genotype the fatty acid percentage represents the mean ± SD of three technical replicates.
