# Supplementary material for: The impact of reducing fatty acid desaturation on the composition and thermal stability of rapeseed oil
Source: Plant Biotechnol J. 2019 Oct 14;18(4):983–91. doi: 10.1111/pbi.13263 (PMC7061866; doi:10.1111/pbi.13263)
Supplement: Supplementary file 7 — Table S4 Total antioxidant capacity of oils subjected to thermal stability testing. [file PBI-18-983-s001.docx]

**Supplementary Table 4. Total antioxidant capacity of oils subjected to thermal stability testing**

| **Line** | **nMol Cu^2+^ reduced** |
| --- | --- |
| Nikita | 36.0 ± 10.2 |
| Maplus | 39.5 ± 13.2 |
| K0472 | 30.8 ± 14.7 |
| K0472-HE | 26.0 ± 10.0 |

For each genotype the TAC concentration represents the mean ± SD of four technical replicates.
