# Supplementary material for: The impact of reducing fatty acid desaturation on the composition and thermal stability of rapeseed oil
Source: Plant Biotechnol J. 2019 Oct 14;18(4):983–91. doi: 10.1111/pbi.13263 (PMC7061866; doi:10.1111/pbi.13263)
Supplement: Supplementary file 8 — Appendix S1 Sequence alignment of amplicons of Bna.FAE1.A8, Bna.FAE1.C3, Bna.FAD2.A5 and Bna.FAD2.C5 copies. [file PBI-18-983-s002.docx]

**Supplementary File 1. Sequence alignment of amplicons of *Bna.FAE1.A8, Bna.FAE1.C3, Bna.FAD2.A5* and *Bna.FAD2.C5* copies.**

The mutations are highlighted in yellow.

***Bna.FAE1.A8* Amplicon**

10 20 30 40 50 60 70 80 90 100

....|....| ....|....| ....|....| ....|....| ....|....| ....|....| ....|....| ....|....| ....|....| ....|....|

**Cabriolet** **CCACTGTATA** **GAGATACATA** **CATGTAAACA** **ATCAAAATAC** **TAGAACACCA** **TTGCATTCTT** **TATTCATAAC** **AAACAAGAGA** **AACATCGTAG** **CCATCAAATT**

**HELP 2-91** **----------** **----------** **----------** **----------** **----------** **----------** **----------** **----------** **----------** **----------**

**HELP 4-87** **----------** **----------** **----------** **----------** **----------** **----------** **----------** **----------** **----------** **----------**

110 120 130 140 150 160 170 180 190 200

....|....| ....|....| ....|....| ....|....| ....|....| ....|....| ....|....| ....|....| ....|....| ....|....|

**Cabriolet** **ATTATAACAA** **ATAAAAAGAA** **ACGAAAGAGA** **GCAAACATCA** **TTTATTAGGA** **CCGACCGTTT** **TGGACACGAG** **TCTCTGACTT** **ACCTGAATCA** **GAATCAATTT**

**HELP 2-91** **----------** **----------** **----------** **----------** **----------** **----------** **----------** **----------** **----------** **----------**

**HELP 4-87** **----------** **----------** **----------** **----------** **----------** **----------** **----------** **----------** **----------** **----------**

210 220 230 240 250 260 270 280 290 300

....|....| ....|....| ....|....| ....|....| ....|....| ....|....| ....|....| ....|....| ....|....| ....|....|

**Cabriolet** **TGACCGGGTA** **TCTGTCGATG** **CAGTGTTCCC** **AAGGACTATT** **TGTCGAAGCT** **TTGACATTGT** **TTAGAGCCAC** **CCAAACTGCA** **CTGTTACACT** **TAAAGCCTGA**

**HELP 2-91** **----------** **----------** **----------** **----------** **----------** **----------** **----------** **----------** **----------** **----------**

**HELP 4-87** **----------** **----------** **----------** **----------** **----------** **----------** **----------** **----------** **----------** **----------**

310 320 330 340 350 360 370 380 390 400

....|....| ....|....| ....|....| ....|....| ....|....| ....|....| ....|....| ....|....| ....|....| ....|....|

**Cabriolet** **CCCTAAAGCA** **ATCTGCCAAA** **CTTTATTACC** **TTTCTTCATC** **CTTCCTTTTG** **CTTCTATGTA** **TGCCAACTCA** **TACCATATTG** **AGCTAGATGA** **AGTGTTTCCA**

**HELP 2-91** **----------** **----------** **----------** **----------** **----------** **----------** **----------** **----------** **----------** **----------**

**HELP 4-87** **----------** **----------** **----------** **----------** **----------** **----------** **----------** **----------** **----------** **----------**

410 420 430 440 450 460 470 480 490 500

....|....| ....|....| ....|....| ....|....| ....|....| ....|....| ....|....| ....|....| ....|....| ....|....|

**Cabriolet** **AATCTATGTA** **ACGTTGATCT** **TGATGCCTCT** **ACATCGATCG** **GTGCTAGGGC** **TAGGTTCTTC** **TCTAGCACAT** **CAATCACGGC** **-TCTGCCTCC** **G-GCATGTAT**

**HELP 2-91** **----------** **----------** **----------** **----------** **----------** **--GGAATTTT** **CCTGTATCAT** **CA----CGGC** **-TCTGCCTCC** **G-GCATGTAT**

**HELP 4-87** **----------** **----------** **----------** **----------** **----------** **--CGAACTGG** **CGGGACAAAT** **CA----CGGA** **GTTTGCCTCC** **GAGCATGTAT**

510 520 530 540 550 560 570 580 590 600

....|....| ....|....| ....|....| ....|....| ....|....| ....|....| ....|....| ....|....| ....|....| ....|....|

**Cabriolet** **ACAAAAATGG** **TCAATAGCAA** **GTTTGAAATC** **CGGGACGTAG** **TAATGTATGA** **TTTTATCTTT** **GAAAAGTTTC** **TTGCCCATGA** **AGGTAACGAA** **AAAAAGAAGT**

**HELP 2-91** **ACAAAAATGG** **TCGATAGCAA** **GTTTGAAGTC** **CGGGACGTAA** **TAATGTTTGA** **TTTTGTCTTT** **GAAAAGTTTC** **TTGCCCATGA** **AGGTAACGAA** **AAAAAGAAGT**

**HELP 4-87** **ACAAAAATGG** **TCAATAGCAA** **GCTTGAAATC** **CGGGACGTAA** **TAATGTTTGA** **TTTTATCTTT** **GAAAAGTTTC** **TTGCCCATGA** **AGGTAACGAA** **AAAAAGAAGT**

610 620 630 640 650 660 670 680 690 700

....|....| ....|....| ....|....| ....|....| ....|....| ....|....| ....|....| ....|....| ....|....| ....|....|

**Cabriolet** **TTCTCGCTTA** **ACGGAAGAAT** **CAACGGACCC** **AACGTTGCTA** **TGTTTTTCTT** **AACCGTTCGA** **CCAGCAACAT** **CGGTTATGTC** **CTTGGACAAA** **CTCACTCCGA**

**HELP 2-91** **TTCTCGCTTA** **ACGGAAGAAT** **CAACGGACCC** **AACGTTGCTA** **TGTTTTTCTT** **AACCGTTCGA** **CCAGCAACAT** **CGGTTATGTC** **CTTGGACAAA** **CTCACTCCGA**

**HELP 4-87** **TTCTCGCTTA** **ACGGAAGAAT** **CAACGGACCC** **AACGTTGCTA** **TGTTTTTCTT** **AACCGTTCGA** **CCAGCAACAT** **CGGTTATGTC** **CTTGGACAAA** **CTCACTCCGA**

710 720 730 740 750 760 770 780 790 800

....|....| ....|....| ....|....| ....|....| ....|....| ....|....| ....|....| ....|....| ....|....| ....|....|

**Cabriolet** **TTTTGCCGTT** **CTCATCGTCT** **CCTTGTTGCA** **CGCAACGAAG** **AGACTTGTCG** **TCAGCTCCGG** **TATGCGTTCG** **AACCGTGTGA** **ACTAGCTCGT** **ACTTGGACCG**

**HELP 2-91** **TTTTGCCGTT** **CTCATCGTCT** **CCTTGTTGCA** **CGCAACGAAA** **AGACTTGTCG** **TCAGCTCCGG** **TATGCGTTCG** **AACCGTGTGA** **ACTAGCTCGT** **ACTTGGACCG**

**HELP 4-87** **TTTTGCCGTT** **CTCATCGTCT** **CCTTGTTGCA** **CGCAACGAAA** **AGACTTGTCG** **TCAGCTCCGG** **TATGCGTTCG** **AACCGTGTGA** **ACTAGCTCGT** **ACTTGGACCG**

810 820 830 840 850 860 870 880 890 900

....|....| ....|....| ....|....| ....|....| ....|....| ....|....| ....|....| ....|....| ....|....| ....|....|

**Cabriolet** **TCTACGATCT** **CCAGGCTTGT** **TGAAGAGCAA** **AATAGCGGCC** **CCACCAACAC** **GGAACAAGCA** **ATTTGAAACC** **ATCATGGACC** **TATTATCACC** **AGCGTAAATG**

**HELP 2-91** **TCTACGATCT** **CCAGGCTTGT** **TGGAGAGCAA** **AATAGCGGCC** **CCACCAACAC** **GGAACAAGCA** **ATTTGAAACC** **ATCATGGACC** **TATTATCACC** **AGCGTAAATG**

**HELP 4-87** **TCTACGATCT** **CCAGGCTTGT** **TGGAGAGCAA** **AATAGCGGCC** **CCACCAACAC** **GGAACAAGCA** **ATTTGAAACC** **ATCATGGACC** **TATTATCACC** **AGCGTAAATG**

910 920 930 940 950 960 970 980 990 1000

....|....| ....|....| ....|....| ....|....| ....|....| ....|....| ....|....| ....|....| ....|....| ....|....|

**Cabriolet** **TTATAAGTGA** **TGTTCTCTGT** **GCTCACCACA** **AGAGCATACG** **TATTTTTATG** **GACATGCAAC** **AAGTCCTTTG** **CTAGATCAAT** **GGCTATAACG** **CCGGCACTAC**

**HELP 2-91** **TTATAAGTGA** **TGTTCTCTGT** **GCTCACCACA** **AGAGCATACG** **TATTTTTATG** **GACATGCAAC** **AAGTCCTTTG** **CTAGATCAAT** **GGCTATAACG** **CCGGCACTAC**

**HELP 4-87** **TTATAAGTGA** **TGTTCTCTGT** **GCTCACCACA** **AGAGCATACG** **TATTTTTATG** **GACATGCAAC** **AAGTCCTTTG** **CTAGATCAAT** **GGCTATAACG** **CCGGCACTAC**

1010 1020 1030 1040 1050 1060 1070 1080 1090 1100

....|....| ....|....| ....|....| ....|....| ....|....| ....|....| ....|....| ....|....| ....|....| ....|....|

**Cabriolet** **AACCCATGCC** **ACCAAGGTTA** **AAGCTTCTTA** **CGTTGCTTCG** **GAGCTTGAAA** **GTGTTAACGA** **CCATCGCGGA** **TAGCGATGGA** **GT-GGAATAA** **ACATGCTTGA**

**HELP 2-91** **AACCCATGCC** **ACCAAGGTTA** **AAGCTTCTTA** **CGTTGCTTCG** **GAGCTTGAAA** **GTGTTAACGA** **CCATCGCGGA** **GAGCGATGGA** **GTTGGATTAA** **ACATGCTTGA**

**HELP 4-87** **AACCCATGCC** **ACCAAGGTTA** **AAGCTTCTTA** **CGTTGCTTCG** **GAGCTTGAAA** **GTGTTAACGA** **CCATCGCGGA** **GAGCGAAGGA** **GTTGGATTAA** **ACATGCTTGA**

1110 1120 1130 1140 1150 1160 1170 1180 1190 1200

....|....| ....|....| ....|....| ....|....| ....|....| ....|....| ....|....| ....|....| ....|....| ....|....|

**Cabriolet** **GTTCCCCCCA** **GG-ATTCCTA** **TCTCTTTAAG** **GGTCAACGTT** **GGTGGTCTTG** **AATGGATTTA** **CAGCCGC-CC** **AATGATAGCT** **TGGTCCGTCC** **CTCCCCGAGC**

**HELP 2-91** **GTTCACCACA** **AGTATACCTA** **TATCTTTA-G** **GGTTAACGTT** **GGTGTTCTTG** **AATAGATTTT** **CTAGCGCACC** **AATGATAACT** **TGCTCCGTCT** **CTTCACGCGC**

**HELP 4-87** **GTTCACCACA** **AGTATACCTA** **TATCTTTA-G** **GGTTAACATT** **GGTGTTCTTG** **AATAGATTTT** **CTAGCGCACC** **AATGATAACT** **TGCTCCGTCT** **CTTCACGCGC**

1210 1220 1230 1240 1250 1260 1270 1280 1290 1300

....|....| ....|....| ....|....| ....|....| ....|....| ....|....| ....|....| ....|....| ....|....| ....|....|

**Cabriolet** **CGCCAAAAAA** **GTTTTCTGGG** **TAGGGACCTG** **GAGGCAGCCC** **CTTGGGCCCG** **TGAATTTCAA** **CGCCTA-ACC** **CGGACGGTTT** **TGAAA-----** **----------**

**HELP 2-91** **CGCCGCAAAA** **GTCTTCCGGG** **GAGGGACCTG** **AAG-CAGCCC** **CTCGGGCCCG** **TGAGTTTCAT** **CGCCTAGACC** **TGAACGTTCT** **TGAATCTTCC** **TCAAGAAGTC**

**HELP 4-87** **CGCCGCAAAA** **GTCTTCCGGG** **GAGGGACCTG** **AAG-CAGCCC** **CTCGGGCCCG** **TGAGTTTCAT** **CGCCTAGACC** **TGAACGTTCT** **TGAATCTTCC** **TCAAGAAGTC**

1310 1320 1330 1340 1350 1360 1370 1380 1390 1400

....|....| ....|....| ....|....| ....|....| ....|....| ....|....| ....|....| ....|....| ....|....| ....|....|

**Cabriolet** **----------** **----------** **----------** **----------** **----------** **----------** **----------** **----------** **----------** **----------**

**HELP 2-91** **AAGCCACGAC** **GAGTCATCGC** **ACGTGCCGTT** **CCGAGAAGGA** **TCAGCTTTT-** **CTTACTTGAT** **AAAAGATATC** **CATGACCTTG** **G-AGATACTT** **-GATCTACAA**

**HELP 4-87** **AAGCCACGAC** **GAGTCATCGC** **ACGTGCCGTT** **CCGAGAAGGA** **TCAGCTTTTT** **CTTACTTGAT** **AAAAGATATC** **CATGACCTTG** **GGAGATACTT** **TGATCTACAA**

1410 1420 1430 1440 1450 1460 1470 1480 1490 1500

....|....| ....|....| ....|....| ....|....| ....|....| ....|....| ....|....| ....|....| ....|....| ....|....|

**Cabriolet** **----------** **----------** **----------** **----------** **----------** **----------** **----------** **----------** **----------** **----------**

**HELP 2-91** **TGCGTTGGTG** **GAAAGGTAGC** **ATGAGTACTC** **AACGAGGTAA** **ACCGGGTTTG** **GGCCGGGTTG** **CGATGTAGAG** **AACCGAACCC** **GAAAACGGTG** **AAG-CAAGAG**

**HELP 4-87** **TGCGTTGGTG** **GAA-GGTAGC** **ATGAGTACTC** **AACGAGGTAA** **ACCGGTTTTG** **GGCCGGGTTG** **CGATGTAGAA** **GAACCGAACC** **GAAAACGGTG** **AAGGCAAGAG**

1510 1520 1530 1540 1550 1560 1570 1580 1590 1600

....|....| ....|....| ....|....| ....|....| ....|....| ....|....| ....|....| ....|....| ....|....| ....|....|

**Cabriolet** **----------** **----------** **----------** **----------** **----------** **----------** **----------** **----------** **----------** **----------**

**HELP 2-91** **TGAGCGATGG** **TTATGAGGTT** **GTGTTGGAGA** **-TAG--ATAG** **TATAAGTGGT** **GA--GATCGT** **CTATGTAGCG** **AATAGGCTTT** **CCGGCGACGA** **TCGCCGTACT**

**HELP 4-87** **TGAGCGATGG** **TTATGAGGTT** **GTGTTGGAGA** **ATAGGAATAG** **TATA-GTGGT** **GAAGAATCGT** **C---------** **----------** **----------** **----------**

1610 1620 1630 1640 1650 1660 1670 1680 1690 1700

....|....| ....|....| ....|....| ....|....| ....|....| ....|....| ....|....| ....|....| ....|....| ....|....|

**Cabriolet** **----------** **----------** **----------** **----------** **----------** **----------** **----------** **----------** **----------** **----------**

**HELP 2-91** **GAAAGAAGCA** **AGGTGGAAAA** **GTGCTATGAC** **GTATGATTAA** **GGACTACGTA** **ACTGTCGTCA** **ATGGCCTCAT** **GTGTGTGTTG** **TTCGGGACAA** **ATATGAGTTA**

**HELP 4-87** **----------** **----------** **----------** **----------** **----------** **----------** **----------** **----------** **----------** **----------**

....|....

**Cabriolet** **---------**

**HELP 2-91** **TAATGCAGC**

**HELP 4-87** **---------**

***Bna.FAE1.C3* Amplicon**

10 20 30 40 50 60 70 80 90 100

....|....| ....|....| ....|....| ....|....| ....|....| ....|....| ....|....| ....|....| ....|....| ....|....|

**Cabriolet** **---------A** **ATTTTTTCTT** **TAAGGATCT-** **CA-AAAAGGG** **TTTTAGGGTT** **AAAGATGGTC** **TAAGAGATAC** **ATACATGTAA** **AACAATCAAA** **TACTAGAACA**

**HELP 2-91** **CGGGAAAAAA** **AGTTTTAGTT** **TAAGTAATC-** **TC-AAAAGGG** **TTTTAGGGTT** **AAAGATGGTC** **TAAGAGATAC** **ATACATGTAA** **AACAATCAAA** **TACTAGAACA**

**HELP 4-87** **----------** **-TGTAGTAAA** **AAGTGATCTG** **CACAAAAGGG** **TTTTAGGGTT** **AAAGATGGTC** **TAAGAGATAC** **ATACATGTAA** **AACAATCAAA** **TACTAGAACA**

110 120 130 140 150 160 170 180 190 200

....|....| ....|....| ....|....| ....|....| ....|....| ....|....| ....|....| ....|....| ....|....| ....|....|

**Cabriolet** **CCGTTGCATT** **CTTTATTCAT** **AACAAACAAG** **AGAAACATCG** **TAGCCATCAA** **ATTATTATAA** **CCAATAAAAA** **GAAACGAAAG** **AGAGCAAACA** **TCATTTATTA**

**HELP 2-91** **CCGTTGCATT** **CTTTATTCAT** **AACAAACAAG** **AGAAACATCG** **TAGCCATCAA** **ATTATTATAA** **CCAATAAAAA** **GAAACGAAAG** **AGAGCAAACA** **TCATTTATTA**

**HELP 4-87** **CCGTTGCATT** **CTTTATTCAT** **AACAAACAAG** **AGAAACATCG** **TAGCCATCAA** **ATTATTATAA** **CCAATAAAAA** **GAAACGAAAG** **AGAGCAAACA** **TCATTTATTA**

210 220 230 240 250 260 270 280 290 300

....|....| ....|....| ....|....| ....|....| ....|....| ....|....| ....|....| ....|....| ....|....| ....|....|

**Cabriolet** **GGACCGACCG** **TTTTGGACAC** **GAGTCTCTGA** **CTTACCTGAA** **TCAGAATCAA** **TTTTAACCGG** **GTATCTGTCG** **ATGCAGTGTT** **CCCAAGGACT** **ATTTG--GAA**

**HELP 2-91** **GGACCGACCG** **TTTTGGACAC** **GAGTCTCTGA** **CTTACCTGAA** **TCAGAATCAA** **TTTTAACCGG** **GTATCTGTCG** **ATGCAGTGTT** **CCCAAGGACT** **ATTTGTTGAA**

**HELP 4-87** **GGACCGACCG** **TTTTGGACAC** **GAGTCTCTGA** **CTTACCTGAA** **TCAGAATCAA** **TTTTAACCGG** **GTATCTGTCG** **ATGCAGTGTT** **CCCAAGGACT** **ATTTGTTGAA**

310 320 330 340 350 360 370 380 390 400

....|....| ....|....| ....|....| ....|....| ....|....| ....|....| ....|....| ....|....| ....|....| ....|....|

**Cabriolet** **GCTTTGACAT** **TGTTTAGAGC** **CACCCAAACT** **GCACTGTTAC** **ACTTAAAGCC** **TGACCCTAAA** **GCAATCTGCC** **AAACTTTATT** **ACCTTTCTTC** **ATCCTTCCTT**

**HELP 2-91** **GCTTTGACAT** **TGTTTAGAGC** **CACCCAAACT** **GCACTGTTAC** **ACTTAAAGCC** **TGACCCTAAA** **GCAATCTGCC** **AAACTTTATT** **ACCTTTCTTC** **ATCCTTCCTT**

**HELP 4-87** **GCTTTGACAT** **TGTTTAGAGC** **CACCCAAACT** **GCACTGTTAC** **ACTTAAAGCC** **TGACCCTAAA** **GCAATCTGCC** **AAACTTTATT** **ACCTTTCTTC** **ATCCTTCCTT**

410 420 430 440 450 460 470 480 490 500

....|....| ....|....| ....|....| ....|....| ....|....| ....|....| ....|....| ....|....| ....|....| ....|....|

**Cabriolet** **TTGCTTCTAT** **GTATGCCAAC** **TCATACCATA** **TTGAGCTAGA** **TGAAGTGTTT** **CCAAATCTAT** **GTAACGTTGA** **TCTTGATGCC** **TCTACATCGA** **TCGGTGCTAG**

**HELP 2-91** **TTGCTTCTAT** **GTATGCCAAC** **TCATACCATA** **TTGAGCTAGA** **TGAAGTGTTT** **CCAAATCTAT** **GTAACGTTGA** **TCTTGATGCC** **TCTACATCGA** **TCGGTGCTAG**

**HELP 4-87** **TTGCTTCTAT** **GTATGCCAAC** **TCATACCATA** **TTGAGCTAGA** **TGAAGTGTTT** **CCAAATCTAT** **GTAACGTTGA** **TCTTGATGCC** **TCTACATCGA** **TCGGTGCTAG**

510 520 530 540 550 560 570 580 590 600

....|....| ....|....| ....|....| ....|....| ....|....| ....|....| ....|....| ....|....| ....|....| ....|....|

**Cabriolet** **GCCTAGGTTC** **TTCTCTAGCA** **CATCAATCAC** **GGCTTTGCCT** **CCGGCATGTA** **TACAAAAATG** **GTCGATAGCA** **AGCTTGAAGT** **CCGGGACGTA** **ATAATGTTTG**

**HELP 2-91** **GCCTAGGTTC** **TTCTCTAGCA** **CATCAATCAC** **GGCTTTGCCT** **CCGGCATGTA** **TACAAAAATG** **GTCGATAGCA** **AGCTTGAAGT** **CCGGGACGTA** **ATAATGTTTG**

**HELP 4-87** **GCCTAGGTTC** **TTCTCTAGCA** **CATCAATCAC** **GGCTTTGCCT** **CCGGCATGTA** **TACAAAAATG** **GTCGATAGCA** **AGCTTGAAGT** **CCGGGACGTA** **ATAATGTTTG**

610 620 630 640 650 660 670 680 690 700

....|....| ....|....| ....|....| ....|....| ....|....| ....|....| ....|....| ....|....| ....|....| ....|....|

**Cabriolet** **ATTTTGTCTT** **TGAAAAGTTT** **CTTGCCCATG** **AAGGTAACGA** **AAAAAAGAAG** **TTTCTCGCTT** **AACGGAAGAA** **TCAACGGACC** **CAGCGTTGCT** **ATGTTTTTCT**

**HELP 2-91** **ATTTTGTCTT** **TGAAAAGTTT** **CTTGCCCATG** **AAGGTAACGA** **AAAAAAGAAG** **TTTCTCGCTT** **AACGGAAGAA** **TCAACGGACC** **CAGCGTTGCT** **ATGTTTTTCT**

**HELP 4-87** **ATTTTGTCTT** **TGAAAAGTTT** **CTTGCCCATG** **AAGGTAACGA** **AAAAAAGAAG** **TTTCTCGCTT** **AACGGAAGAA** **TCAACGGACC** **CAGCGTTGCT** **ATGTTTTTCT**

710 720 730 740 750 760 770 780 790 800

....|....| ....|....| ....|....| ....|....| ....|....| ....|....| ....|....| ....|....| ....|....| ....|....|

**Cabriolet** **TAACCGTTCG** **ACCAGCAACA** **TCGGTTATGT** **CCTTGGACAA** **ACTCACTCCG** **GTTTTGCCGT** **TCTCATCGTC** **TCCTTGTTGC** **ACGCAACGAA** **AAGACTTGTC**

**HELP 2-91** **TAACCGTTCG** **ACCAGCAACA** **TCGGTTATGT** **CCTTGGACAA** **ACTCACTCCG** **GTTTTGCCGT** **TCTCATCGTC** **TCCTTGTTGC** **ACGCAACGAA** **AAGACTTGTC**

**HELP 4-87** **TAACCGTTCG** **ACCAGCAACA** **TCGGTTATGT** **CCTTGGACAA** **ACTCACTCCG** **GTTTTGCCGT** **TCTCATCGTC** **TCCTTGTTGC** **ACGCAACGAA** **AAGACTTGTC**

810 820 830 840 850 860 870 880 890

....|....| ....|....| ....|....| ....|....| ....|....| ....|....| ....|....| ....|....| ....|....| ...

**Cabriolet** **GTCAGCTCCG** **GTATGCGTTC** **GAACCGTGTG** **AACTAGCTCG** **TACTTGGACC** **GTCTACGATC** **TCTAGGCTTG** **TTGGAGAGCA** **AAA-TAGCGG** **GCA**

**HELP 2-91** **GTCAGCTCCG** **GTATGCGTTC** **GAACCGTGTG** **AACTAGCTCG** **TACTTGGACC** **GTCTACGATC** **TCTAGGCTTG** **TTGGAGAGCA** **AAAATAGCCG** **GCA**

**HELP 4-87** **GTCAGCTCCG** **GTATGCGTTC** **GAACCGTGTG** **AACTAGCTCG** **TACTTGGACC** **GTCTACGATC** **TCTAGGCTTG** **TTGGAAAAGC** **AAAATAGCCG** **GCA**

***Bna.FAD2.A5* Amplicon**

10 20 30 40 50 60 70 80 90 100

....|....| ....|....| ....|....| ....|....| ....|....| ....|....| ....|....| ....|....| ....|....| ....|....|

**Maplus**  **AAATCAAGCG** **CGTACCCTGC** **GAGACACCGC** **CCTTCACTGT** **CGGAGAACTC** **AAGAAAGCAA** **TCCCACCGCA** **CTGTTTCAAA** **CGCTCGATCC** **CTCGCTCTTT**

**HELP 2-91** **-TATCT-GTG** **AGTACCCTGC** **GAGACACCGC** **CCTTCACTGT** **CGGAGAACTC** **AAGAAAGCAA** **TCCCACCGCA** **CTGTTTCAAA** **CGCTCGATCC** **CTCGCTCTTT**

**HELP 4-87** **-----AGACG** **CGTACCCTGC** **GAGACACCGC** **CCTTCACTGT** **CGGAGAACTC** **AAGAAAGCAA** **TCCCACCGCA** **CTGTTTCAAA** **CGCTCGATCC** **CTCGCTCTTT**

110 120 130 140 150 160 170 180 190 200

....|....| ....|....| ....|....| ....|....| ....|....| ....|....| ....|....| ....|....| ....|....| ....|....|

**Maplus**  **CTCCTACCTC** **ATCTGGGACA** **TCATCATAGC** **CTCCTGCTTC** **TACTACGTCG** **CCACCACTTA** **CTTCCCTCTC** **CTCCCTCACC** **CTCTCTCCTA** **CTTCGCCTGG**

**HELP 2-91** **CTCCTACCTC** **ATCTGGGACA** **TCATCATAGC** **CTCCTGCTTC** **TACTACGTCG** **CCACCA-TTA** **CTTCCCTCTC** **CTCCCTCACC** **CTCTCTCCTA** **CTTCGCCTGG**

**HELP 4-87** **CTCCTACCTC** **ATCTGGGACA** **TCATCATAGC** **CTCCTGCTTC** **TACTACGTCG** **CCACCA-TTA** **CTTCCCTCTC** **CTCCCTCACC** **CTCTCTCCTA** **CTTCGCCTGG**

210 220 230 240 250 260 270 280 290 300

....|....| ....|....| ....|....| ....|....| ....|....| ....|....| ....|....| ....|....| ....|....| ....|....|

**Maplus**  **CCTCTCTACT** **GGGCCTGCCA** **GGGCTGCGTC** **CTAACCGGCG** **TCTGGGTCAT** **AGCCCACGAG** **TGCGGCCACC** **ACGCCTTCAG** **CGACTACCAG** **TGGCTGGACG**

**HELP 2-91** **CCTCTCTACT** **GGGCCTGCCA** **GGGCTGCGTC** **CTAACCGGCG** **TCTGGGTCAT** **AGCCCACGAG** **TGCGGCCACC** **ACGCCTTCAG** **CGACTACCAG** **TGGCTGGACG**

**HELP 4-87** **CCTCTCTACT** **GGGCCTGCCA** **GGGCTGCGTC** **CTAACCGGCG** **TCTGGGTCAT** **AGCCCACGAG** **TGCGGCCACC** **ACGCCTTCAG** **CGACTACCAG** **TGGCTGGACG**

310 320 330 340 350 360 370 380 390 400

....|....| ....|....| ....|....| ....|....| ....|....| ....|....| ....|....| ....|....| ....|....| ....|....|

**Maplus**  **ACACCGTCGG** **CCTCATCTTC** **CACTCCTTCC** **TCCTCGTCCC** **TTACTTCTCC** **TGGAAGTACA** **GTCATCGACG** **CCACCATTCC** **AACACTGGCT** **CCCTCGAGAG**

**HELP 2-91** **ACACCGTCGG** **CCTCATCTTC** **CACTCCTTCC** **TCCTCGTCCC** **TTACTTCTCC** **TGGAAGTACA** **GTCATCGACG** **CCACCATTCC** **AACACTGGCT** **CCCTCGAGAG**

**HELP 4-87** **ACACCGTCGG** **CCTCATCTTC** **CACTCCTTCC** **TCCTCGTCCC** **TTACTTCTCC** **TGGAAGTACA** **GTCATCGACG** **CCACCATTCC** **AACACTGGCT** **CCCTCGAGAG**

410 420 430 440 450 460 470 480 490 500

....|....| ....|....| ....|....| ....|....| ....|....| ....|....| ....|....| ....|....| ....|....| ....|....|

**Maplus**  **AGACGAAGTG** **TTTGTCCCCA** **AGAAGAAGTC** **AGACATCAAG** **TGGTACGGCA** **AGTACCTCAA** **CAACCCTTTG** **GGACGCACCG** **TGATGTTAAC** **GGTTCAGTTC**

**HELP 2-91** **AGACGAAGTG** **TTTGTCCCCA** **AGAAGAAGTC** **AGACATCAAG** **TGGTACGGCA** **AGTACCTCAA** **CAACCCTTTG** **GGACGCACCG** **TGATGTTAAC** **GGTTCAGTTC**

**HELP 4-87** **AGACGAAGTG** **TTTGTCCCCA** **AGAAGAAGTC** **AGACATCAAG** **TGGTACGGCA** **AGTACCTCAA** **CAACCCTTTG** **GGACGCACCG** **TGATGTTAAC** **GGTTCAGTTC**

510 520 530 540 550 560 570 580 590 600

....|....| ....|....| ....|....| ....|....| ....|....| ....|....| ....|....| ....|....| ....|....| ....|....|

**Maplus**  **ACTCTCGGCT** **GGCCTTTGTA** **CTTAGCCTTC** **AACGTCTCGG** **GGAGACCTTA** **CGACGGCGGC** **TTCGCTTGCC** **ATTTCCACCC** **CAACGCTCCC** **ATCTACAACG**

**HELP 2-91** **ACTCTCGGCT** **GGCCTTTGTA** **CTTAGCCTTC** **AACGTCTCGG** **GGAGACCTTA** **CGACGGCGGC** **TTCGCTTGCC** **ATTTCCACCC** **CAACGCTCCC** **ATCTACAACG**

**HELP 4-87** **ACTCTCGGCT** **GGCCTTTGTA** **CTTAGCCTTC** **AACGTCTCGG** **GGAGACCTTA** **CGACGGCGGC** **TTCGCTTGCC** **ATTTCCACCC** **CAACGCTCCC** **ATCTACAACG**

610 620 630 640 650 660 670 680 690 700

....|....| ....|....| ....|....| ....|....| ....|....| ....|....| ....|....| ....|....| ....|....| ....|....|

**Maplus**  **ACCGTGAGCG** **TCTCCAGATA** **TACATCTCCG** **ACGCTGGCAT** **CCTCGCCGTC** **TGCTACGGTC** **TCTACCGCTA** **CGCTGCTGTC** **CAAGGAGTTG** **CCTCGATGGT**

**HELP 2-91** **ACCGTGAGCG** **TCTCCAGATA** **TACATCTCCG** **ACGCTGGCAT** **CCTCGCCGTC** **TGCTACGGTC** **TCTACCGCTA** **CGCTGCTGTC** **CAAGGAGTTG** **CCTCGATGGT**

**HELP 4-87** **ACCGTGAGCG** **TCTCCAGATA** **TACATCTCCG** **ACGCTGGCAT** **CCTCGCCGTC** **TGCTACGGTC** **TCTACCGCTA** **CGCTGCTGTC** **CAAGGAGTTG** **CCTCGATGGT**

710 720 730 740 750 760 770 780 790 800

....|....| ....|....| ....|....| ....|....| ....|....| ....|....| ....|....| ....|....| ....|....| ....|....|

**Maplus**  **CTGCTTCTAC** **GGAGTTCCTC** **TTCTGATTGT** **CAACGGGTTC** **TTAGTTTTGA** **TCACTTACTT** **GCAGCACACG** **CATCCTTCCC** **TGCCTCACTA** **TGACTCGTCT**

**HELP 2-91** **CTGCTTCTAC** **GGAGTTCCTC** **TTCTGATTGT** **CAACGGGTTC** **TTAGTTTTGA** **TCACTTACTT** **GCAGCACACG** **CATCCTTCCC** **TGCCTCACTA** **TGACTCGTCT**

**HELP 4-87** **CTGCTTCTAC** **GGAGTTCCTC** **TTCTGATTGT** **CAACGGGTTC** **TTAGTTTTGA** **TCACTTACTT** **GCAGCACACG** **CATCCTTCCC** **TGCCTCACTA** **TGACTCGTCT**

810 820 830 840 850 860 870 880 890 900

....|....| ....|....| ....|....| ....|....| ....|....| ....|....| ....|....| ....|....| ....|....| ....|....|

**Maplus**  **GAGTGGGATT** **GGTTGAGGGG** **AGCTTTGGCC** **ACCGTTGACA** **GAGACTACGG** **AATCTTGAAC** **AAGGTCTTCC** **ACAATATCAC** **GGACACGCAC** **GTGGCGCATC**

**HELP 2-91** **GAGTGGGATT** **GGTTGAGGGG** **AGCTTTGGCC** **ACCGTTGACA** **GAGACTACGG** **AATCTTGAAC** **AAGGTCTTCC** **ACAATATCAC** **GGACACGCAC** **GTGGCGCATC**

**HELP 4-87** **GAGTGGGATT** **GGTTGAGGGG** **AGCTTTGGCC** **ACCGTTGACA** **GAGACTACGG** **AATCTTGAAC** **AAGGTCTTCC** **ACAATATCAC** **GGACACGCAC** **GTGGCGCATC**

910 920 930 940 950 960 970 980 990 1000

....|....| ....|....| ....|....| ....|....| ....|....| ....|....| ....|....| ....|....| ....|....| ....|....|

**Maplus**  **ACCTGTTCTC** **GACCATGCCG** **CATTATCATG** **CGATGGAAGC** **TACGAAGGCG** **ATAAAGCCGA** **TACTGGGAGA** **GTATTATCAG** **TTCGATGGGA** **CGCC-GGTGG**

**HELP 2-91** **ACCTGTTCTC** **GACCATGCCG** **CATTATCATG** **CGATGGAAGC** **TACGAAGGCG** **ATAAAGCCGA** **TACTGGGAGA** **GTATTATCAG** **TTCGATGGGA** **CGCCCGGTGG**

**HELP 4-87** **ACCTGTTCTC** **GACCATGCCG** **CATTATCATG** **CGATGGAAGC** **TACGAAGGCG** **ATAAAGCCGA** **TACTGGGAGA** **GTATTATCAG** **TTCGATGGGA** **CGCC-GGTGG**

1010 1020 1030 1040 1050 1060 1070 1080 1090 1100

....|....| ....|....| ....|....| ....|....| ....|....| ....|....| ....|....| ....|....| ....|....| ....|....|

**Maplus**  **TT--AAGCGA** **TGTGGAGGGA** **GGCGAAGGAG** **TGTATCTATG** **TGGAACCGGG** **ACAGGCAAGT** **GGAGAAGAAA** **GGTGGTGTCT** **GGTACATAAA** **TATATATTTT**

**HELP 2-91** **TTTAAGGCGA** **TGTGGAGGGA** **G-CGAG--AG** **TGTATCTATG** **TGGA--CCGG** **ACAGGCA-GT** **G--AGAGAAA** **GGTGTGTTCT** **GGTACACTTA** **AAATTTTTTT**

**HELP 4-87** **TT--AAGCGA** **TGTGGAAGGA** **GGCGAAG-AG** **TGTATCTATG** **TGGAACCCGG** **ACAGGCAAGT** **G-AGAAAGAA** **AGTGGTGTTT** **CTGGTTACAA** **CATAAAATTT**

1110

....|....| .

**Maplus**  **TAGTAGGAGG** **G**

**HELP 2-91** **TAAGAGAGG-** **-**

**HELP 4-87** **--ATTGAAGG** **A**

***Bna.FAD2.C5* Amplicon**

10 20 30 40 50 60 70 80 90 100

....|....| ....|....| ....|....| ....|....| ....|....| ....|....| ....|....| ....|....| ....|....| ....|....|

**Maplus**  **CATAAGGGCG** **TACCGTGCGA** **GACACCGCCC** **TTCACTGTCG** **GAGAACTCAA** **GAAAGCAATC** **CCACCGCACT** **GTTTCGAACG** **CTCGATCCCT** **CGCTCTTTCT**

**HELP 2-91** **--------AG** **TACCCTGCGA** **GACACCGCCC** **TTCACTGTCG** **GAGAACTCAA** **GAAAGCAATC** **CCACCGCACT** **GTTTCAAACG** **CTCGATCCCT** **CGCTCTTTCT**

**HELP 4-87** **----ACAGCG** **TACCGTGCGA** **GACACCGCCC** **TTCACTGTCG** **GAGAACTCAA** **GAAAGCAATC** **CCACCGCACT** **GTTTCAAACG** **CTCGATCCCT** **CGCTCTTTCT**

110 120 130 140 150 160 170 180 190 200

....|....| ....|....| ....|....| ....|....| ....|....| ....|....| ....|....| ....|....| ....|....| ....|....|

**Maplus**  **CCTACCTCAT** **CTGGGACATC** **ATCATAGCCT** **CCTGCTTCTA** **CTACGTCGCC** **ACCACTTACT** **TCCCTCTCCT** **CCCTCACCCT** **CTCTCCTACT** **TCGCCTGGCC**

**HELP 2-91** **CCTACCTCAT** **CTGGGACATC** **ATCATAGCCT** **CCTGCTTCTA** **CTACGTCGCC** **ACCACTTACT** **TCCCTCTCCT** **CCCTCACCCT** **CTCTCCTACT** **TCGCCTGGCC**

**HELP 4-87** **CCTACCTCAT** **CTGGGACATC** **ATCATAGCCT** **CCTGCTTCTA** **CTACGTCGCC** **ACCACTTACT** **TCCCTCTCCT** **CCCTCACCCT** **CTCTCCTACT** **TCGCCTGGCC**

210 220 230 240 250 260 270 280 290 300

....|....| ....|....| ....|....| ....|....| ....|....| ....|....| ....|....| ....|....| ....|....| ....|....|

**Maplus**  **TCTCTACTGG** **GCCTGCCAAG** **GGTGCGTCCT** **AACCGGCGTC** **TGGGTCATAG** **CCCACGAGTG** **CGGCCACCAC** **GCCTTCAGCG** **ACTACCAGTG** **GCTTGACGAC**

**HELP 2-91** **TCTCTACTGG** **GCCTGCCAAG** **GGTACGTCCT** **AACCGGCGTC** **TGGGTCATAG** **CCCACGAGTG** **CGGCCACCAC** **GCCTTCAGCG** **ACTACCAGTG** **GCTTGACGAC**

**HELP 4-87** **TCTCTACTGG** **GCCTGCCAAG** **GGTGCGTCCT** **AACCGGCGTC** **TGGGTCATAG** **CCCACGAGTG** **CGGCCACCAC** **GCCTTCAGCG** **ACTACCAGTG** **GCTTGACGAC**

310 320 330 340 350 360 370 380 390 400

....|....| ....|....| ....|....| ....|....| ....|....| ....|....| ....|....| ....|....| ....|....| ....|....|

**Maplus**  **ACCGTCGGTC** **TCATCTTCCA** **CTCCTTCCTC** **CTCGTCCCTT** **ACTTCTCCTG** **GAAGTACAGT** **CATCGACGCC** **ACCATTCCAA** **CACTGGCTCC** **CTCGAGAGAG**

**HELP 2-91** **ACCGTCGGTC** **TCATCTTCCA** **CTCCTTCCTC** **CTCGTCCCTT** **ACTTCTCCTG** **GAAGTACAGT** **CATCGACGCC** **ACCATTCCAA** **CACTGGCTCC** **CTCGAGAGAG**

**HELP 4-87** **ACCGTCGGTC** **TCATCTTCCA** **CTCCTTCCTC** **CTCGTCCCTT** **ACTTCTCCTG** **GAAGTACAGT** **CATCGACGCC** **ACCATTCCAA** **CACTGGCTCC** **CTCGAGAGAG**

410 420 430 440 450 460 470 480 490 500

....|....| ....|....| ....|....| ....|....| ....|....| ....|....| ....|....| ....|....| ....|....| ....|....|

**Maplus**  **ACGAAGTGTT** **TGTCCCCAAG** **AAGAAGTCAG** **ACATCAAGTG** **GTACGGCAAG** **TACCTCAACA** **ACCCTTTGGG** **ACGCACCGTG** **ATGTTAACGG** **TTCAGTTCAC**

**HELP 2-91** **ACGAAGTGTT** **TGTCCCCAAG** **AAGAAGTCAG** **ACATCAAGTG** **GTACGGCAAG** **TACCTCAACA** **ACCCTTTGGG** **ACGCACCGTG** **ATGTTAACGG** **TTCAGTTCAC**

**HELP 4-87** **ACGAAGTGTT** **TGTCCCCAAG** **AAGAAGTCAG** **ACATCAAGTG** **GTACGGCAAG** **TACCTCAACA** **ACCCTTTGGG** **ACGCACCGTG** **ATGTTAACGG** **TTCAGTTCAC**

510 520 530 540 550 560 570 580 590 600

....|....| ....|....| ....|....| ....|....| ....|....| ....|....| ....|....| ....|....| ....|....| ....|....|

**Maplus**  **TCTCGGCTGG** **CCGTTGTACT** **TAGCCTTCAA** **CGTCTCGGGA** **AGACCTTACG** **ACGGCGGCTT** **CGCTTGCCAT** **TTCCACCCCA** **ACGCTCCCAT** **CTACAACGAC**

**HELP 2-91** **TCTCGGCTGG** **CCGTTGTACT** **TAGCCTTCAA** **CGTCTCGGGA** **AGACCTTACG** **ACGGCGGCTT** **CGCTTGCCAT** **TTCCACCCCA** **ACGCTCCCAT** **CTACAACGAC**

**HELP 4-87** **TCTCGGCTGG** **CCGTTGTACT** **TAGCCTTCAA** **CGTCTCGGGA** **AGACCTTACG** **ACGGCGGCTT** **CGCTTGCCAT** **TTCCACCCCA** **ACGCTCCCAT** **CTACAACGAC**

610 620 630 640 650 660 670 680 690 700

....|....| ....|....| ....|....| ....|....| ....|....| ....|....| ....|....| ....|....| ....|....| ....|....|

**Maplus**  **CGCGAGCGTC** **TCCAGATATA** **CATCTCCGAC** **GCTGGCATCC** **TCGCCGTCTG** **CTACGGTCTC** **TTCCGTTACG** **CCGCCGCGCA** **GGGAGTGGCC** **TCGATGGTCT**

**HELP 2-91** **CGCGAGCGTC** **TCCAGATATA** **CATCTCCGAC** **GCTGGCATCC** **TCGCCGTCTG** **CTACGGTCTC** **TTCCGTTACG** **CCGCCGCGCA** **GGGAGTGGCC** **TCGATGGTCT**

**HELP 4-87** **CGCGAGCGTC** **TCCAGATATA** **CATCTCCGAC** **GCTGGCATCC** **TCGCCGTCTG** **CTACGATCTC** **TTCCGTTACG** **CCGCCGCGCA** **GGGAGTGGCC** **TCGATGGTCT**

710 720 730 740 750 760 770 780 790 800

....|....| ....|....| ....|....| ....|....| ....|....| ....|....| ....|....| ....|....| ....|....| ....|....|

**Maplus**  **GCTTCTACGG** **AGTCCCGCTT** **CTGATTGTCA** **ATGGTTTCCT** **CGTGTTGATC** **ACTTACTTGC** **AGCACACGCA** **TCCTTCCCTG** **CCTCACTACG** **ATTCGTCCGA**

**HELP 2-91** **GCTTCTACGG** **AGTCCCGCTT** **CTGATTGTCA** **ATGGTTTCCT** **CGTGTTGATC** **ACTTACTTGC** **AGCACACGCA** **TCCTTCCCTG** **CCTCACTACG** **ATTCGTCCGA**

**HELP 4-87** **GCTTCTACGG** **AGTCCCGCTT** **CTGATTGTCA** **ATGGTTTCCT** **CGTGTTGATC** **ACTTACTTGC** **AGCACACGCA** **TCCTTCCCTG** **CCTCACTACG** **ATTCGTCCGA**

810 820 830 840 850 860 870 880 890 900

....|....| ....|....| ....|....| ....|....| ....|....| ....|....| ....|....| ....|....| ....|....| ....|....|

**Maplus**  **GTGGGATTGG** **TTGAGGGGAG** **CTTTGGCTAC** **CGTTGACAGA** **GACTACGGAA** **TCTTGAACAA** **GGTCTTCCAC** **AATATTACCG** **ACACGCACGT** **GGCGCATCAT**

**HELP 2-91** **GTGGGATTGG** **TTGAGGGGAG** **CTTTGGCTAC** **CGTTGACAGA** **GACTACGGAA** **TCTTGAACAA** **GGTCTTCCAC** **AATATTACCG** **ACACGCACGT** **GGCGCATCAT**

**HELP 4-87** **GTGGGATTGG** **TTGAGGGGAG** **CTTTGGCTAC** **CGTTGACAGA** **GACTACGGAA** **TCTTGAACAA** **GGTCTTCCAC** **AATATTACCG** **ACACGCACGT** **GGCGCATCAT**

910 920 930 940 950 960 970 980 990 1000

....|....| ....|....| ....|....| ....|....| ....|....| ....|....| ....|....| ....|....| ....|....| ....|....|

**Maplus**  **CTGTTCTCCA** **CGATGCCGCA** **TTATCACGCG** **ATGGAAGCTA** **CCAAAGGCGA** **TAAAGCCGAT** **ACTGGGAGAG** **TATTATCAGT** **TCGATGGGAC** **GCCGGTGGTT**

**HELP 2-91** **CTGTTCTCCA** **CGATGCCGCA** **TTATCACGCG** **ATGGAAGCTA** **CCAA-GGCGA** **TAAAGCCGAT** **ACTGGGAGAG** **TATTATCAGT** **TCGATGGGAC** **GCCGGTGGTT**

**HELP 4-87** **CTGTTCTCCA** **CGATGCCGCA** **TTATCACGCG** **ATGGAAGCTA** **CCAA-GGCGA** **TAAAGCCGAT** **ACTGGGAGAG** **TATTATCAGT** **TCGATGGGAC** **GCCGGTGTTT**

1010 1020 1030 1040 1050 1060 1070 1080 1090 1100

....|....| ....|....| ....|....| ....|....| ....|....| ....|....| ....|....| ....|....| ....|....| ....|....|

**Maplus**  **AAG-CGATGT** **G-AGGGA-GG** **CGAA--GAGT** **GTTATCTATG** **--TGAACCGG** **GACAGGC---** **AGTGAGAA--** **GAAAGGTG-T** **GTTCTGGTAC** **A--CAT--AG**

**HELP 2-91** **AAGGCGATGT** **GGAGGGA-GG** **CGAA--GGAG** **TGTATCTATG** **--TGGAACCG** **GACAGGC---** **AGTGAGAA--** **GAAAGGTGGT** **GTTCTGGTAC** **AA-CAATAAG**

**HELP 4-87** **AAGGCGATGT** **GGAGGGAAGG** **CGAAAGGAGT** **TGTATCTATT** **GTTGAACCCG** **GACAGGCCAA** **AGTGAAGAAG** **GAAAGGTGTG** **TTTCTTGTAC** **AAACCAATAG**

1110 1120 1130 1140 1150 1160 1170 1180

....|....| ....|....| ....|....| ....|....| ....|....| ....|....| ....|....| ....|....| ....|....

**Maplus**  **TTAT-GAGAT** **ATTGATGATG** **TTGAAGAACA** **AAGAAGATAT** **T-GTCACGAA** **CCTTTCTCTT** **GCTCTCCGCT** **GGGGTCCGCT** **CTGGAATC-**

**HELP 2-91** **TTAT-GAGAT** **AT-GATGATG** **GTGAAGAACA** **AAGAAGATAT** **TTGTCACGA-** **CCTTTCTCTT** **GCTGTCTCTC** **TGGGGCGCCT** **CTGTAAAAA**

**HELP 4-87** **TTATTGAGAT** **AT-GATGATG** **GTGTAAGAAC** **CAGAAGAATT** **TGTTCACGAA** **CCTTTCTCTG** **GCTGTTCTTC** **TGGGTCCGTC** **CTTGAAAAT**
