# Supplementary material for: The impact of reducing fatty acid desaturation on the composition and thermal stability of rapeseed oil
Source: Plant Biotechnol J. 2019 Oct 14;18(4):983–91. doi: 10.1111/pbi.13263 (PMC7061866; doi:10.1111/pbi.13263)
Supplement: Supplementary file 9 — Appendix S2 Detailed fatty acid composition of HELP F4 seeds. For each sample, the fatty acid percentage represents the mean of three technical replicates. [file PBI-18-983-s003.docx]

Supplementary Table 2. Detailed fatty acid composition (percentages) of HELP lines (F_4_ seeds) and parental controls.

For each sample, the fatty acid % by weight represent the mean value of three technical replicates.

| **Line** | **C16:0** | **C16:1** | **C18:0** | **C18:1** | **C18:2** | **C18:3** | **C20:0** | **C20:1** | **C22:0** | **C22:1** | **C24:0** | **C24:1** |  |
| --- | --- | --- | --- | --- | --- | --- | --- | --- | --- | --- | --- | --- | --- |
| **‘Maplus x K0472' HELP F_4_ seeds** | | | | | | | | | | | | | |
| **2-91-1** | 3.1 | 0.0 | 0.6 | 28.8 | 2.3 | 4.0 | 0.4 | 8.1 | 0.0 | 51.8 | 0.0 | 0.8 |  |
| **2-91-2** | 2.3 | 0.2 | 0.6 | 25.0 | 1.9 | 4.0 | 0.6 | 7.1 | 0.4 | 56.9 | 0.1 | 0.9 |  |
| **2-91-3** | 2.0 | 0.2 | 0.6 | 27.1 | 1.3 | 2.9 | 0.6 | 8.6 | 0.4 | 55.4 | 0.1 | 0.8 |  |
| **2-91-4** | 2.4 | 0.2 | 0.6 | 25.6 | 1.7 | 3.9 | 0.6 | 7.5 | 0.4 | 56.0 | 0.0 | 0.8 |  |
| **2-91-5** | 2.2 | 0.2 | 0.6 | 23.9 | 2.1 | 4.6 | 0.7 | 6.1 | 0.5 | 58.0 | 0.1 | 1.0 |  |
| **2-91-6** | 2.2 | 0.2 | 0.7 | 26.7 | 1.4 | 3.0 | 0.6 | 7.2 | 0.4 | 56.8 | 0.0 | 0.9 |  |
| **2-91-7** | 2.4 | 0.2 | 0.6 | 27.8 | 2.3 | 3.6 | 0.5 | 10.5 | 0.3 | 50.7 | 0.0 | 1.0 |  |
| **2-91-8** | 2.7 | 0.4 | 0.6 | 26.7 | 1.8 | 4.4 | 0.6 | 6.0 | 0.3 | 55.5 | 0.1 | 0.9 |  |
| **2-91-9** | 2.2 | 0.2 | 0.8 | 27.8 | 1.3 | 2.7 | 0.7 | 9.2 | 0.4 | 53.9 | 0.0 | 0.8 |  |
| **2-91-10** | 3.2 | 0.4 | 0.8 | 29.0 | 1.9 | 3.3 | 0.7 | 10.6 | 0.4 | 48.9 | 0.0 | 0.7 |  |
| **'Maplus x K0047' HELP F_4_ seeds** | | | | | | | | | | | | | |
| **4-87-1** | 2.3 | 0.2 | 0.7 | 23.1 | 3.5 | 5.6 | 0.7 | 7.7 | 0.5 | 54.7 | 0.2 | 0.9 |  |
| **4-87-2** | 2.1 | 0.2 | 0.8 | 25.6 | 2.6 | 4.5 | 0.6 | 9.3 | 0.4 | 53.1 | 0.0 | 0.8 |  |
| **4-87-3** | 2.1 | 0.2 | 0.8 | 24.6 | 2.5 | 4.6 | 0.7 | 7.6 | 0.5 | 55.6 | 0.0 | 0.8 |  |
| **4-87-4** | 2.2 | 0.2 | 0.8 | 23.7 | 3.3 | 5.3 | 0.8 | 9.5 | 0.4 | 53.1 | 0.0 | 0.7 |  |
| **4-87-5** | 2.1 | 0.2 | 0.7 | 24.8 | 2.4 | 4.5 | 0.7 | 7.7 | 0.5 | 55.6 | 0.0 | 0.9 |  |
| **4-87-6** | 2.2 | 0.2 | 1.0 | 27.0 | 2.2 | 4.1 | 0.7 | 10.9 | 0.4 | 50.5 | 0.1 | 0.8 |  |
| **4-87-7** | 2.2 | 0.3 | 0.8 | 24.3 | 3.0 | 5.0 | 0.7 | 8.8 | 0.5 | 53.7 | 0.1 | 0.8 |  |
| **4-87-8** | 2.2 | 0.2 | 0.8 | 24.5 | 3.0 | 4.8 | 0.7 | 9.2 | 0.4 | 53.3 | 0.1 | 0.8 |  |
| **4-87-9** | 2.2 | 0.3 | 0.7 | 24.0 | 2.7 | 4.9 | 0.7 | 7.1 | 0.5 | 56.0 | 0.2 | 0.9 |  |
| **4-87-10** | 2.1 | 0.2 | 0.8 | 26.9 | 2.0 | 4.0 | 0.6 | 9.9 | 0.4 | 52.1 | 0.1 | 0.8 |  |
| **Parental Controls – Mutants and HEAR** | | | | | | | | | | | | | |
| **K0472-1** | 3.7 | 0.4 | 1.2 | 84.0 | 2.0 | 4.6 | 0.7 | 2.0 | 0.6 | 0.2 | 0.5 | 0.0 |  |
| **K0472-2** | 3.4 | 0.3 | 1.2 | 83.4 | 2.3 | 5.1 | 0.8 | 2.1 | 0.7 | 0.1 | 0.5 | 0.0 |  |
| **K0472-3** | 3.5 | 0.3 | 0.8 | 87.0 | 1.8 | 3.7 | 0.4 | 1.8 | 0.3 | 0.0 | 0.2 | 0.1 |  |
| **K0472-4** | 3.5 | 0.2 | 0.8 | 86.1 | 2.0 | 4.2 | 0.4 | 1.9 | 0.3 | 0.0 | 0.2 | 0.2 |  |
| **K0472-5** | 3.5 | 0.2 | 0.8 | 87.0 | 1.9 | 3.6 | 0.4 | 1.8 | 0.3 | 0.0 | 0.2 | 0.2 |  |
| **K0472-6** | 3.3 | 0.0 | 1.3 | 86.8 | 2.4 | 3.7 | 0.7 | 1.7 | 0.0 | 0.0 | 0.0 | 0.0 |  |
| **K0472-7** | 3.3 | 0.0 | 1.5 | 87.6 | 1.5 | 3.8 | 0.7 | 1.5 | 0.0 | 0.0 | 0.0 | 0.0 |  |
| **K0472-8** | 3.4 | 0.0 | 1.5 | 87.8 | 1.5 | 3.9 | 0.3 | 1.6 | 0.0 | 0.0 | 0.0 | 0.0 |  |
| **K0472-9** | 3.3 | 0.0 | 1.5 | 87.7 | 1.4 | 3.6 | 0.6 | 1.6 | 0.2 | 0.0 | 0.0 | 0.0 |  |
| **K0472-10** | 3.4 | 0.0 | 1.5 | 86.9 | 1.5 | 4.2 | 0.7 | 1.7 | 0.0 | 0.0 | 0.0 | 0.0 |  |
| **K0047-1** | 4.4 | 0.4 | 1.0 | 78.6 | 3.5 | 8.1 | 0.8 | 2.1 | 0.7 | 0.0 | 0.5 | 0.1 |  |
| **K0047-2** | 3.4 | 0.3 | 1.6 | 85.5 | 2.1 | 4.2 | 0.8 | 1.4 | 0.7 | 0.0 | 0.2 | 0.0 |  |
| **K0047-3** | 3.6 | 0.3 | 0.9 | 83.9 | 2.8 | 5.6 | 0.5 | 1.7 | 0.3 | 0.0 | 0.2 | 0.2 |  |
| **K0047-4** | 3.7 | 0.2 | 0.9 | 84.4 | 2.6 | 5.0 | 0.5 | 1.8 | 0.3 | 0.0 | 0.2 | 0.2 |  |
| **K0047-5** | 4.1 | 0.2 | 1.0 | 82.0 | 3.4 | 7.0 | 0.3 | 1.7 | 0.2 | 0.0 | 0.2 | 0.1 |  |
| **K0047-6** | 4.1 | 0.2 | 1.0 | 82.0 | 3.4 | 7.0 | 0.3 | 1.7 | 0.2 | 0.0 | 0.2 | 0.1 |  |
| **K0047-7** | 4.1 | 0.2 | 1.0 | 82.0 | 3.4 | 7.0 | 0.3 | 1.7 | 0.2 | 0.0 | 0.2 | 0.1 |  |
| **K0047-8** | 4.1 | 0.2 | 1.0 | 82.0 | 3.4 | 7.0 | 0.3 | 1.7 | 0.2 | 0.0 | 0.2 | 0.1 |  |
| **K0047-9** | 4.2 | 0.1 | 1.2 | 80.0 | 4.1 | 7.3 | 0.3 | 2.0 | 0.5 | 0.2 | 0.2 | 0.1 |  |
| **K0047-10** | 4.2 | 0.1 | 1.2 | 80.0 | 4.1 | 7.3 | 0.3 | 2.0 | 0.5 | 0.2 | 0.2 | 0.1 |  |
| **Maplus-1** | 4.2 | 0.3 | 0.9 | 11.5 | 13.7 | 9.9 | 0.7 | 10.9 | 0.5 | 46.4 | 0.0 | 0.4 |  |
| **Maplus-2** | 4.3 | 0.3 | 0.9 | 11.1 | 14.2 | 10.1 | 0.7 | 10.5 | 0.5 | 46.3 | 0.1 | 0.5 |  |
| **Maplus-3** | 4.9 | 0.4 | 0.7 | 10.2 | 17.3 | 9.9 | 0.5 | 7.2 | 0.5 | 46.8 | 0.1 | 0.9 |  |
| **Maplus-4** | 4.1 | 0.2 | 0.8 | 14.7 | 16.2 | 9.0 | 0.7 | 9.0 | 0.5 | 42.7 | 0.2 | 1.0 |  |
| **Maplus-5** | 3.9 | 0.2 | 0.8 | 11.1 | 16.3 | 9.0 | 0.6 | 8.4 | 0.6 | 47.5 | 0.2 | 0.9 |  |
| **Maplus-6** | 3.5 | 0.2 | 0.7 | 11.9 | 15.6 | 8.3 | 0.6 | 8.1 | 0.6 | 49.1 | 0.2 | 0.8 |  |
| **Maplus-7** | 3.8 | 0.2 | 0.7 | 11.0 | 16.6 | 9.1 | 0.5 | 7.1 | 0.6 | 48.9 | 0.1 | 0.9 |  |
| **Mapls-8** | 4.3 | 0.2 | 0.7 | 9.9 | 15.1 | 8.3 | 0.5 | 6.6 | 0.5 | 52.5 | 0.1 | 0.5 |  |
| **Maplus-9** | 4.2 | 0.2 | 1.0 | 15.3 | 16.2 | 7.3 | 0.7 | 9.5 | 0.6 | 43.5 | 0.2 | 0.8 |  |
| **Maplus-10** | 5.1 | 0.3 | 0.8 | 13.1 | 17.9 | 7.4 | 0.7 | 8.6 | 0.6 | 43.8 | 0.2 | 0.8 |  |
