# Supplementary material for: The impact of reducing fatty acid desaturation on the composition and thermal stability of rapeseed oil
Source: Plant Biotechnol J. 2019 Oct 14;18(4):983–91. doi: 10.1111/pbi.13263 (PMC7061866; doi:10.1111/pbi.13263)
Supplement: Supplementary file 10 — Appendix S3 Detailed fatty acid composition of HELP F5 seeds. [file PBI-18-983-s004.docx]

**Supplementary File 3. Detailed fatty acid composition of HELP F_5_ seeds**

Fatty acid composition (% by weight) of 169 individual HELP lines (K0472-HE) and control Maplus was measured with two technical replicates each and the following values are represented as percentages.

| **Line** | **C16:0** | **C16:1** | **C18:0** | **C18:1** | **C18:2** | **C18:3** | **C20:0** | **C20:1** | **C22:0** | **C22:1** |
| --- | --- | --- | --- | --- | --- | --- | --- | --- | --- | --- |
| Maplus | 3.8 | 0.1 | 0.5 | 18.4 | 15.1 | 9.2 | 0.4 | 3.7 | 0.3 | 48.6 |
| **K0472-HE** |  |  |  |  |  |  |  |  |  |  |
| 2-91-3-2 | 2.0 | 0.3 | 0.0 | 28.0 | 1.2 | 2.7 | 0.6 | 9.0 | 0.0 | 56.3 |
| 2-91-3-3 | 2.1 | 0.3 | 0.4 | 29.1 | 1.1 | 2.4 | 0.7 | 10.9 | 0.2 | 53.0 |
| 2-91-3-4 | 1.9 | 0.2 | 0.7 | 27.7 | 1.2 | 2.8 | 0.6 | 8.0 | 0.0 | 57.1 |
| 2-91-3-5 | 1.9 | 0.3 | 0.4 | 27.6 | 1.2 | 2.9 | 0.6 | 8.1 | 0.2 | 57.0 |
| 2-91-3-6 | 2.0 | 0.3 | 0.8 | 27.3 | 1.3 | 2.9 | 0.7 | 8.1 | 0.4 | 56.1 |
| 2-91-3-7 | 1.9 | 0.3 | 0.4 | 28.1 | 1.2 | 2.7 | 0.6 | 9.3 | 0.6 | 55.0 |
| 2-91-3-8 | 2.1 | 0.3 | 0.7 | 29.7 | 1.2 | 2.8 | 0.7 | 8.2 | 0.2 | 54.1 |
| 2-91-3-9 | 2.4 | 0.2 | 1.0 | 32.1 | 1.1 | 2.1 | 0.8 | 14.0 | 0.4 | 46.2 |
| 2-91-3-10 | 2.2 | 0.3 | 0.9 | 29.9 | 1.2 | 2.4 | 0.7 | 10.4 | 0.0 | 52.1 |
| 2-91-3-11 | 2.2 | 0.3 | 1.0 | 30.7 | 1.1 | 2.4 | 0.7 | 12.2 | 0.0 | 49.6 |
| 2-91-3-12 | 2.2 | 0.3 | 0.8 | 29.9 | 1.1 | 2.5 | 0.6 | 10.2 | 0.0 | 52.6 |
| 2-91-3-13 | 2.2 | 0.3 | 1.0 | 30.5 | 1.0 | 2.1 | 0.7 | 13.8 | 0.3 | 48.2 |
| 2-91-3-14 | 2.1 | 0.3 | 0.9 | 29.3 | 1.1 | 2.3 | 0.8 | 11.2 | 0.2 | 52.0 |
| 2-91-3-15 | 2.0 | 0.3 | 0.8 | 29.1 | 1.0 | 2.3 | 0.7 | 10.2 | 0.4 | 53.3 |
| 2-91-3-16 | 1.9 | 0.0 | 0.6 | 27.4 | 1.4 | 3.1 | 0.6 | 8.1 | 0.0 | 57.0 |
| 2-91-3-17 | 2.1 | 0.3 | 0.9 | 29.4 | 1.0 | 2.2 | 0.7 | 10.9 | 0.0 | 52.7 |
| 2-91-3-18 | 2.1 | 0.3 | 0.8 | 29.0 | 1.1 | 2.4 | 0.7 | 10.5 | 0.2 | 53.1 |
| 2-91-3-19 | 2.0 | 0.3 | 0.8 | 28.1 | 1.0 | 2.4 | 0.7 | 8.6 | 0.4 | 55.8 |
| 2-91-3-20 | 4.0 | 0.1 | 0.8 | 29.0 | 1.1 | 2.5 | 0.7 | 10.3 | 0.0 | 51.7 |
| 2-91-3-21 | 2.0 | 0.2 | 0.8 | 29.2 | 1.1 | 2.5 | 0.6 | 10.7 | 0.0 | 53.0 |
| 2-91-3-22 | 2.0 | 0.3 | 0.8 | 28.7 | 1.1 | 2.4 | 0.7 | 10.0 | 0.4 | 53.8 |
| 2-91-3-23 | 2.1 | 0.3 | 0.8 | 28.6 | 1.1 | 2.5 | 0.6 | 9.0 | 0.4 | 54.8 |
| 2-91-3-24 | 2.0 | 0.3 | 0.8 | 28.3 | 1.1 | 2.5 | 0.7 | 9.2 | 0.4 | 54.8 |
| 2-91-9-1 | 2.0 | 0.4 | 0.6 | 27.4 | 1.3 | 3.0 | 0.7 | 7.2 | 0.5 | 57.0 |
| 2-91-9-2 | 2.0 | 0.3 | 0.7 | 28.0 | 1.2 | 2.5 | 0.6 | 8.8 | 0.5 | 55.6 |
| 2-91-9-3 | 2.2 | 0.3 | 0.9 | 28.7 | 1.1 | 2.3 | 0.7 | 10.6 | 0.4 | 53.0 |
| 2-91-9-4 | 2.1 | 0.3 | 0.6 | 28.1 | 1.4 | 3.0 | 0.6 | 6.6 | 0.6 | 56.8 |
| 2-91-9-5 | 2.3 | 0.0 | 0.0 | 28.4 | 1.2 | 2.7 | 0.0 | 8.8 | 0.0 | 56.7 |
| 2-91-9-6 | 2.1 | 0.3 | 0.7 | 28.7 | 1.3 | 2.7 | 0.6 | 8.5 | 0.4 | 54.8 |
| 2-91-9-7 | 2.0 | 0.3 | 0.6 | 27.3 | 1.4 | 3.1 | 0.7 | 6.9 | 0.5 | 57.3 |
| 2-91-9-8 | 2.0 | 0.3 | 0.7 | 28.9 | 1.4 | 2.9 | 0.6 | 8.1 | 0.5 | 54.8 |
| 2-91-9-9 | 2.1 | 0.3 | 0.7 | 28.0 | 1.4 | 3.0 | 0.6 | 9.0 | 0.4 | 54.7 |
| 2-91-9-10 | 2.0 | 0.3 | 0.6 | 27.4 | 1.2 | 2.9 | 0.6 | 7.3 | 0.4 | 57.3 |
| 2-91-9-11 | 2.7 | 0.5 | 0.6 | 26.6 | 1.6 | 3.3 | 0.6 | 6.3 | 0.5 | 57.5 |
| 2-91-9-12 | 2.0 | 0.3 | 0.7 | 28.2 | 1.3 | 2.7 | 0.6 | 8.7 | 0.4 | 55.1 |
| 2-91-9-14 | 2.1 | 0.3 | 0.7 | 28.3 | 1.3 | 2.7 | 0.7 | 8.0 | 0.5 | 55.7 |
| 2-91-9-15 | 2.1 | 0.4 | 0.8 | 28.0 | 1.4 | 2.8 | 0.7 | 8.5 | 0.5 | 55.0 |
| 2-91-9-16 | 2.1 | 0.3 | 0.7 | 29.1 | 1.3 | 2.8 | 0.6 | 8.2 | 0.5 | 54.6 |
| 2-91-9-18 | 2.3 | 0.3 | 0.8 | 28.3 | 1.3 | 2.7 | 0.7 | 9.2 | 0.4 | 54.3 |
| 2-91-9-21 | 2.1 | 0.3 | 0.7 | 28.7 | 1.3 | 2.7 | 0.6 | 9.7 | 0.5 | 53.6 |
| 2-91-9-22 | 2.1 | 0.4 | 0.7 | 28.2 | 1.6 | 2.8 | 0.7 | 7.3 | 0.5 | 55.9 |
| 2-91-9-23 | 1.9 | 0.3 | 0.6 | 27.6 | 1.3 | 3.1 | 0.6 | 7.2 | 0.9 | 56.5 |
| 2-91-9-24 | 2.1 | 0.3 | 0.7 | 27.7 | 1.2 | 2.6 | 0.7 | 9.0 | 0.4 | 55.2 |
| 2-91-9-25 | 2.0 | 0.3 | 0.6 | 26.8 | 1.4 | 3.3 | 0.6 | 6.9 | 0.5 | 57.7 |
| **Line** | **C16:0** | **C16:1** | **C18:0** | **C18:1** | **C18:2** | **C18:3** | **C20:0** | **C20:1** | **C22:0** | **C22:1** |
| 2-91-9-26 | 2.0 | 0.3 | 0.8 | 27.1 | 1.3 | 3.8 | 0.7 | 7.4 | 0.5 | 54.5 |
| 2-91-9-27 | 2.6 | 0.4 | 0.6 | 27.8 | 1.4 | 3.0 | 0.6 | 7.8 | 0.4 | 55.5 |
| 2-91-9-28 | 2.1 | 0.3 | 0.8 | 28.8 | 1.2 | 2.5 | 0.7 | 9.4 | 0.5 | 53.8 |
| 2-91-9-29 | 2.0 | 0.3 | 0.7 | 28.2 | 1.3 | 2.9 | 0.7 | 7.3 | 0.5 | 56.3 |
| 2-91-9-30 | 2.2 | 0.3 | 0.8 | 28.7 | 1.2 | 2.5 | 0.7 | 8.4 | 0.4 | 54.9 |
| 2-91-9-31 | 2.0 | 0.3 | 0.7 | 27.9 | 1.3 | 2.8 | 0.7 | 6.7 | 0.5 | 57.0 |
| 2-91-9-32 | 2.3 | 0.3 | 0.9 | 28.5 | 1.5 | 3.0 | 0.7 | 10.2 | 0.4 | 52.4 |
| 2-91-9-33 | 2.4 | 0.4 | 0.9 | 27.2 | 1.9 | 3.8 | 0.8 | 10.6 | 0.4 | 51.7 |
| 2-91-9-34 | 2.0 | 0.3 | 0.7 | 28.9 | 1.2 | 2.5 | 0.7 | 7.7 | 0.5 | 55.6 |
| 2-91-9-35 | 2.1 | 0.3 | 0.7 | 27.7 | 1.5 | 2.9 | 0.6 | 8.3 | 0.5 | 55.6 |
| 2-91-4-1 | 2.1 | 0.3 | 0.8 | 29.0 | 1.2 | 2.7 | 0.7 | 8.6 | 0.5 | 54.3 |
| 2-91-4-2 | 2.1 | 0.3 | 0.6 | 27.0 | 1.6 | 3.7 | 0.6 | 8.2 | 0.4 | 54.9 |
| 2-91-4-3 | 2.4 | 0.3 | 0.9 | 27.0 | 1.9 | 4.2 | 0.8 | 10.5 | 0.4 | 51.7 |
| 2-91-4-4 | 2.1 | 0.4 | 0.8 | 28.6 | 1.2 | 2.6 | 0.7 | 8.8 | 0.5 | 54.4 |
| 2-91-4-5 | 2.2 | 0.3 | 0.7 | 29.0 | 1.1 | 2.6 | 0.7 | 9.4 | 0.9 | 53.3 |
| 2-91-4-6 | 2.3 | 0.4 | 0.7 | 27.6 | 1.3 | 2.9 | 0.7 | 6.5 | 0.5 | 57.1 |
| 2-91-4-7 | 2.2 | 0.3 | 0.7 | 28.2 | 1.4 | 2.8 | 0.7 | 8.8 | 0.3 | 54.6 |
| 2-91-2-1 | 2.0 | 0.4 | 0.6 | 27.8 | 1.5 | 3.4 | 0.6 | 6.8 | 0.5 | 56.6 |
| 2-91-2-2 | 2.2 | 0.3 | 0.8 | 27.7 | 1.5 | 3.3 | 0.8 | 9.1 | 0.5 | 53.8 |
| 2-91-2-3 | 2.4 | 0.4 | 0.7 | 27.4 | 1.5 | 3.3 | 0.7 | 7.6 | 0.5 | 55.6 |
| 2-91-2-4 | 2.2 | 0.3 | 0.7 | 26.0 | 1.9 | 4.2 | 0.6 | 7.7 | 0.5 | 55.9 |
| 2-91-2-5 | 2.2 | 0.3 | 0.9 | 27.7 | 1.7 | 3.4 | 0.7 | 10.5 | 0.4 | 52.2 |
| 2-91-2-6 | 2.2 | 0.3 | 0.7 | 25.5 | 2.2 | 4.5 | 0.6 | 7.0 | 0.5 | 56.6 |
| 2-91-2-7 | 2.5 | 0.3 | 0.7 | 27.1 | 1.9 | 3.8 | 0.6 | 8.4 | 0.3 | 54.5 |
| 2-91-2-8 | 2.2 | 0.3 | 0.7 | 25.4 | 2.1 | 4.5 | 0.6 | 7.3 | 0.5 | 56.5 |
| 2-91-2-9 | 2.2 | 0.3 | 0.7 | 26.2 | 1.7 | 4.1 | 0.7 | 7.4 | 0.5 | 56.4 |
| 2-91-2-10 | 2.1 | 0.3 | 0.8 | 26.3 | 1.7 | 4.0 | 0.7 | 7.0 | 0.5 | 56.8 |
| 2-91-2-11 | 2.3 | 0.4 | 0.7 | 27.6 | 1.7 | 3.1 | 0.6 | 7.7 | 0.5 | 55.7 |
| 2-91-2-12 | 2.1 | 0.3 | 0.6 | 24.6 | 2.2 | 5.0 | 0.5 | 5.9 | 0.6 | 57.4 |
| 2-91-2-13 | 2.1 | 0.4 | 0.8 | 26.5 | 1.8 | 3.9 | 0.6 | 6.6 | 0.5 | 56.5 |
| 2-91-2-14 | 2.0 | 0.3 | 0.9 | 28.2 | 1.2 | 2.8 | 0.7 | 8.6 | 1.4 | 54.0 |
| 2-91-2-15 | 2.0 | 0.3 | 0.6 | 24.9 | 2.2 | 4.8 | 0.6 | 6.7 | 0.5 | 57.5 |
| 2-91-2-16 | 2.3 | 0.4 | 0.7 | 25.4 | 2.2 | 4.5 | 0.7 | 7.0 | 0.6 | 56.2 |
| 2-91-2-17 | 2.1 | 0.3 | 1.1 | 27.8 | 1.6 | 3.6 | 0.6 | 7.9 | 0.7 | 54.5 |
| 2-91-2-20 | 2.0 | 0.3 | 0.7 | 26.7 | 1.8 | 3.8 | 0.6 | 8.1 | 0.6 | 55.5 |
| 2-91-2-21 | 2.3 | 0.3 | 0.8 | 27.4 | 1.7 | 3.4 | 0.7 | 10.2 | 0.4 | 52.8 |
| 2-91-2-22 | 2.2 | 0.3 | 0.7 | 26.1 | 2.1 | 4.2 | 0.6 | 6.7 | 0.6 | 56.8 |
| 2-91-2-23 | 2.1 | 0.3 | 0.7 | 26.1 | 1.9 | 4.1 | 0.6 | 7.0 | 0.6 | 56.8 |
| 2-91-2-24 | 2.3 | 0.3 | 0.6 | 25.9 | 1.8 | 4.3 | 0.6 | 7.6 | 0.4 | 56.4 |
| 2-91-2-25 | 2.3 | 0.3 | 0.7 | 25.5 | 2.1 | 4.4 | 0.6 | 7.0 | 0.0 | 57.2 |
| 2-91-2-26 | 2.4 | 0.3 | 0.7 | 27.1 | 1.8 | 3.6 | 0.6 | 8.6 | 0.1 | 54.9 |
| 2-91-5-1 | 2.3 | 0.3 | 0.9 | 23.0 | 3.0 | 5.7 | 0.7 | 6.6 | 0.5 | 57.3 |
| 2-91-5-2 | 2.2 | 0.3 | 0.7 | 24.3 | 2.1 | 4.5 | 0.8 | 6.1 | 0.6 | 58.5 |
| 2-91-5-3 | 3.4 | 0.7 | 0.8 | 24.8 | 2.1 | 4.1 | 0.8 | 7.3 | 0.6 | 55.8 |
| 2-91-5-4 | 2.1 | 0.3 | 0.8 | 23.9 | 2.3 | 5.0 | 0.7 | 6.2 | 0.7 | 58.1 |
| 2-91-5-5 | 2.5 | 0.3 | 0.8 | 25.3 | 2.1 | 4.1 | 0.8 | 7.3 | 0.6 | 56.1 |
| 2-91-5-6 | 2.1 | 0.2 | 0.8 | 24.7 | 2.3 | 4.6 | 0.8 | 6.5 | 1.0 | 57.1 |
| 2-91-5-7 | 2.2 | 0.3 | 0.8 | 25.5 | 1.9 | 4.3 | 0.8 | 6.7 | 0.6 | 57.0 |
| 2-91-5-8 | 2.2 | 0.3 | 0.8 | 25.3 | 0.1 | 4.7 | 0.7 | 7.7 | 0.6 | 57.9 |
| 2-91-5-9 | 2.5 | 0.0 | 0.0 | 29.4 | 2.0 | 3.9 | 0.0 | 9.0 | 0.0 | 53.4 |
| **Line** | **C16:0** | **C16:1** | **C18:0** | **C18:1** | **C18:2** | **C18:3** | **C20:0** | **C20:1** | **C22:0** | **C22:1** |
| 2-91-5-10 | 2.2 | 0.3 | 0.7 | 23.9 | 2.3 | 5.0 | 0.8 | 5.9 | 0.7 | 58.5 |
| 2-91-5-11 | 2.1 | 0.3 | 0.8 | 25.2 | 2.2 | 4.6 | 0.8 | 7.7 | 0.6 | 55.8 |
| 2-91-5-12 | 2.1 | 0.3 | 0.6 | 24.8 | 2.2 | 5.0 | 0.7 | 6.6 | 0.6 | 57.2 |
| 2-91-5-13 | 2.2 | 0.3 | 0.8 | 24.9 | 2.2 | 4.4 | 0.8 | 7.5 | 0.6 | 56.5 |
| 2-91-5-14 | 2.1 | 0.3 | 0.8 | 25.2 | 2.1 | 4.6 | 0.8 | 7.4 | 0.6 | 56.1 |
| 2-91-5-16 | 2.3 | 0.2 | 1.0 | 24.8 | 2.3 | 4.8 | 0.8 | 8.6 | 0.5 | 54.6 |
| 2-91-5-17 | 2.2 | 0.3 | 0.7 | 24.9 | 2.6 | 4.8 | 0.7 | 7.3 | 0.6 | 56.1 |
| 2-91-5-18 | 2.1 | 0.3 | 0.8 | 24.5 | 2.4 | 4.7 | 0.8 | 6.3 | 0.7 | 57.7 |
| 2-91-5-19 | 1.8 | 0.2 | 0.9 | 25.1 | 2.3 | 4.9 | 0.8 | 8.1 | 0.6 | 55.4 |
| 2-91-5-20 | 2.1 | 0.3 | 0.9 | 24.6 | 2.3 | 4.5 | 0.7 | 6.5 | 0.7 | 57.4 |
| 2-91-5-21 | 2.1 | 0.2 | 0.7 | 23.7 | 2.5 | 5.1 | 0.7 | 6.0 | 0.6 | 58.3 |
| 2-91-5-22 | 2.1 | 0.3 | 0.7 | 24.0 | 2.4 | 4.9 | 0.8 | 6.3 | 0.6 | 58.1 |
| 2-91-5-23 | 2.1 | 0.3 | 0.7 | 22.8 | 2.5 | 5.4 | 0.7 | 6.0 | 0.6 | 59.0 |
| 2-91-5-24 | 2.2 | 0.3 | 0.8 | 23.9 | 2.2 | 4.8 | 0.8 | 6.6 | 0.6 | 57.9 |
| 2-91-5-25 | 2.1 | 0.3 | 0.8 | 23.4 | 2.5 | 5.0 | 0.7 | 6.4 | 0.6 | 58.4 |
| 2-91-5-26 | 2.2 | 0.3 | 1.4 | 25.1 | 1.9 | 4.2 | 0.8 | 8.6 | 0.6 | 55.0 |
| 2-91-5-27 | 2.3 | 0.3 | 1.1 | 25.5 | 2.0 | 4.3 | 0.8 | 9.6 | 0.6 | 53.8 |
| 2-91-5-28 | 2.2 | 0.3 | 1.1 | 23.8 | 2.1 | 4.9 | 0.8 | 6.8 | 0.6 | 57.7 |
| 2-91-5-29 | 2.0 | 0.3 | 0.9 | 24.4 | 2.1 | 4.6 | 0.7 | 5.9 | 0.7 | 58.5 |
| 2-91-5-30 | 2.1 | 0.3 | 0.9 | 24.6 | 2.1 | 4.4 | 0.8 | 6.7 | 0.7 | 57.6 |
| 2-91-5-31 | 2.3 | 0.3 | 0.9 | 24.2 | 2.1 | 4.6 | 0.8 | 8.1 | 0.0 | 56.8 |
| 2-91-5-32 | 2.0 | 0.2 | 0.7 | 25.7 | 1.8 | 3.7 | 0.7 | 6.4 | 0.6 | 58.3 |
| 2-91-5-33 | 2.0 | 0.2 | 0.8 | 24.8 | 2.1 | 4.8 | 0.7 | 5.9 | 0.6 | 58.2 |
| 2-91-5-34 | 2.2 | 0.3 | 1.0 | 25.1 | 2.1 | 4.2 | 0.9 | 7.9 | 0.6 | 55.9 |
| 2-91-5-35 | 2.1 | 0.3 | 0.8 | 24.4 | 2.4 | 4.7 | 0.8 | 6.6 | 0.6 | 57.4 |
| 2-91-5-36 | 2.2 | 0.3 | 0.9 | 25.3 | 2.1 | 4.5 | 0.8 | 8.3 | 0.6 | 55.2 |
| 2-91-5-37 | 2.1 | 0.3 | 0.8 | 25.0 | 2.1 | 4.4 | 0.8 | 6.8 | 0.6 | 57.2 |
| 2-91-6-1 | 1.9 | 0.2 | 0.8 | 27.8 | 1.3 | 2.6 | 0.7 | 7.7 | 0.5 | 56.6 |
| 2-91-6-2 | 1.9 | 0.3 | 0.9 | 27.3 | 1.2 | 2.7 | 0.7 | 7.2 | 0.6 | 57.2 |
| 2-91-6-3 | 1.9 | 0.3 | 0.7 | 26.9 | 1.5 | 3.0 | 0.7 | 5.9 | 0.7 | 58.6 |
| 2-91-6-4 | 1.9 | 0.3 | 0.7 | 26.9 | 1.5 | 2.9 | 0.8 | 5.7 | 0.7 | 58.7 |
| 2-91-6-5 | 1.9 | 0.3 | 0.7 | 26.8 | 1.4 | 3.1 | 0.7 | 6.5 | 0.7 | 58.1 |
| 2-91-6-6 | 1.6 | 0.2 | 0.6 | 27.3 | 1.1 | 3.2 | 0.5 | 6.2 | 0.5 | 58.7 |
| 2-91-6-7 | 1.8 | 0.2 | 0.8 | 26.7 | 1.4 | 3.0 | 0.7 | 6.2 | 0.7 | 58.6 |
| 2-91-6-8 | 2.5 | 0.4 | 0.9 | 27.4 | 1.2 | 2.6 | 0.8 | 7.5 | 0.6 | 56.2 |
| 2-91-6-9 | 2.0 | 0.3 | 0.8 | 26.8 | 1.4 | 2.9 | 0.7 | 7.3 | 0.6 | 57.5 |
| 2-91-6-10 | 1.9 | 0.3 | 0.8 | 26.9 | 1.3 | 2.9 | 0.8 | 6.7 | 0.6 | 58.1 |
| 2-91-6-11 | 2.0 | 0.3 | 0.6 | 26.4 | 1.5 | 3.0 | 0.6 | 6.0 | 0.6 | 59.2 |
| 2-91-6-13 | 1.9 | 0.2 | 0.7 | 26.9 | 1.4 | 3.0 | 0.7 | 7.0 | 0.6 | 57.8 |
| 2-91-6-14 | 2.0 | 0.4 | 1.2 | 27.2 | 1.2 | 2.4 | 0.8 | 7.4 | 0.6 | 56.8 |
| 2-91-6-15 | 2.0 | 0.3 | 0.8 | 27.3 | 1.4 | 2.8 | 0.7 | 6.7 | 0.6 | 57.5 |
| 2-91-6-16 | 2.0 | 0.3 | 1.2 | 26.9 | 1.3 | 2.8 | 0.7 | 7.3 | 0.6 | 57.0 |
| 2-91-6-17 | 2.1 | 0.3 | 0.9 | 27.3 | 1.3 | 2.6 | 0.8 | 7.0 | 0.6 | 57.3 |
| 2-91-6-18 | 1.9 | 0.2 | 0.6 | 26.0 | 1.5 | 3.4 | 0.6 | 5.8 | 0.6 | 59.5 |
| 2-91-6-19 | 2.0 | 0.3 | 1.3 | 26.7 | 1.3 | 2.9 | 0.7 | 7.3 | 0.5 | 57.3 |
| 2-91-6-20 | 2.0 | 0.3 | 1.1 | 26.5 | 1.3 | 2.8 | 0.8 | 6.7 | 0.6 | 58.2 |
| 2-91-6-21 | 2.1 | 0.3 | 1.2 | 26.6 | 1.3 | 2.6 | 0.8 | 6.4 | 0.6 | 58.2 |
| 2-91-6-22 | 2.0 | 0.3 | 1.3 | 25.8 | 1.3 | 2.9 | 0.7 | 5.9 | 0.6 | 59.2 |
| 2-91-6-23 | 2.3 | 0.3 | 1.5 | 26.6 | 1.2 | 2.5 | 0.8 | 7.4 | 0.5 | 57.0 |
| 2-91-6-24 | 2.2 | 0.3 | 1.3 | 26.8 | 1.2 | 2.4 | 0.8 | 7.3 | 0.5 | 57.4 |
| **Line** | **C16:0** | **C16:1** | **C18:0** | **C18:1** | **C18:2** | **C18:3** | **C20:0** | **C20:1** | **C22:0** | **C22:1** |
| 2-91-6-25 | 2.3 | 0.3 | 1.4 | 26.2 | 1.2 | 2.4 | 0.8 | 8.0 | 2.6 | 55.1 |
| 2-91-6-26 | 2.0 | 0.3 | 1.3 | 25.9 | 1.3 | 2.9 | 0.7 | 6.1 | 0.6 | 59.1 |
| 2-91-6-27 | 2.1 | 0.4 | 2.1 | 26.0 | 1.4 | 2.7 | 0.8 | 6.2 | 0.7 | 58.0 |
| 2-91-6-28 | 2.4 | 0.4 | 1.3 | 27.3 | 1.1 | 2.2 | 0.8 | 8.9 | 0.7 | 55.3 |
| 2-91-6-29 | 2.2 | 0.3 | 1.0 | 25.9 | 1.3 | 2.8 | 0.6 | 6.0 | 0.7 | 59.4 |
| 2-91-6-30 | 2.0 | 0.3 | 1.2 | 26.5 | 1.2 | 2.6 | 0.9 | 7.1 | 0.5 | 57.9 |
| 2-91-6-31 | 2.2 | 0.2 | 1.0 | 25.4 | 1.4 | 3.3 | 0.7 | 5.5 | 0.6 | 59.7 |
| 2-91-6-32 | 2.1 | 0.3 | 0.7 | 25.8 | 1.5 | 3.0 | 0.8 | 6.5 | 0.4 | 59.0 |
| 2-91-6-33 | 2.9 | 0.0 | 0.0 | 26.7 | 1.7 | 3.5 | 0.0 | 7.5 | 0.0 | 57.7 |
| 2-91-6-34 | 2.2 | 0.3 | 0.6 | 24.4 | 1.9 | 4.0 | 0.9 | 6.4 | 0.7 | 57.9 |
| 2-91-6-36 | 2.8 | 0.4 | 1.3 | 24.3 | 2.5 | 4.5 | 0.7 | 6.8 | 0.6 | 56.0 |
| 2-91-6-37 | 2.3 | 0.3 | 0.9 | 24.9 | 1.9 | 3.4 | 0.8 | 6.9 | 1.2 | 57.5 |
| 2-91-6-38 | 2.2 | 0.4 | 1.1 | 26.6 | 1.3 | 2.7 | 1.1 | 8.4 | 0.5 | 56.0 |
| 2-91-6-39 | 2.4 | 0.4 | 1.1 | 22.6 | 2.7 | 4.8 | 0.7 | 5.6 | 0.6 | 59.1 |
| 2-91-6-40 | 2.3 | 0.4 | 1.9 | 24.8 | 2.1 | 4.1 | 0.7 | 8.2 | 0.5 | 54.8 |
| 2-91-6-41 | 2.1 | 0.3 | 1.5 | 26.4 | 1.3 | 2.9 | 0.7 | 7.6 | 0.6 | 56.9 |
| 2-91-6-42 | 2.9 | 1.9 | 1.6 | 22.4 | 2.5 | 4.3 | 0.7 | 5.2 | 1.0 | 57.5 |
| 2-91-6-43 | 2.5 | 0.3 | 1.0 | 24.3 | 2.5 | 4.4 | 0.5 | 7.4 | 1.2 | 56.0 |
| 2-91-6-44 | 2.5 | 0.3 | 1.1 | 24.0 | 2.2 | 4.3 | 0.6 | 7.0 | 0.5 | 57.4 |
| 2-91-6-45 | 2.5 | 0.4 | 1.0 | 25.3 | 2.0 | 3.4 | 0.7 | 6.5 | 0.7 | 57.7 |
| 2-91-6-46 | 2.3 | 0.3 | 1.2 | 23.6 | 2.2 | 5.1 | 0.6 | 7.6 | 0.8 | 56.5 |
| 2-91-6-47 | 2.7 | 0.5 | 0.9 | 22.7 | 2.9 | 5.1 | 1.0 | 6.9 | 0.9 | 56.8 |
| 2-91-6-48 | 2.4 | 0.2 | 1.3 | 26.7 | 1.6 | 3.0 | 0.7 | 7.9 | 0.4 | 56.0 |
| 2-91-6-49 | 2.8 | 0.4 | 1.0 | 24.4 | 2.4 | 3.9 | 0.8 | 6.8 | 1.4 | 56.0 |
| 2-91-6-50 | 2.6 | 0.2 | 1.3 | 24.3 | 2.3 | 3.9 | 1.1 | 6.8 | 0.3 | 57.3 |
